# Data-driven prediction of lipophilic drug solubility in supercritical CO_2_ using an adaptive ensemble learning architecture

**DOI:** 10.3389/fmed.2026.1780240

**Published:** 2026-05-13

**Authors:** Arwa Sultan Alqahtani, Mahboubeh Pishnamazi

**Affiliations:** 1 Department of Chemistry, College of Science, Imam Mohammad Ibn Saud Islamic University (IMSIU), Riyadh, Saudi Arabia; 2 Institute of Research and Development, Duy Tan University, Da Nang, Vietnam; 3 School of Engineering and Technology, Duy Tan University, Da Nang, Vietnam

**Keywords:** boosting algorithms, ensemble learning, lipophilic drugs, optimization, solubility prediction, supercritical carbon dioxide

## Abstract

**Introduction:**

Predicting how lipophilic pharmaceuticals dissolve in supercritical carbon dioxide (SC-CO_2_) remains a central challenge for process development, largely because experimental measurements are time-consuming and traditional modeling approaches often fail to generalize across chemically diverse compounds.

**Methods:**

This study examines the SC-CO_2_ solubility of four representative drugs Sirolimus, Tacrolimus, Rifampin, and Teriflunomide and introduces an adaptive computational framework designed to improve predictive power across varied molecular structures. The approach combines multiple boosting-based regressors within an ensemble scheme that incorporates a DST-inspired aggregation step and a weighted integration mechanism. Model hyperparameters are optimized using a bio- inspired Electric Eel Foraging strategy. Reliability of the final model was evaluated through repeated cross-validation, comparative statistical tests, and uncertainty estimation.

**Results and discussion:**

The resulting ensemble achieved high predictive accuracy (*R*^2^ = 0.99, RMSE = 0.089) with consistent performance across the investigated compounds. By integrating molecular descriptors, thermodynamic features, and process conditions within a unified ensemble framework, the approach provides a practical computational tool for supporting solubility estimation in SC-CO_2_ systems.

## 1 Introduction

### 1.1 Study background

This technology, Supercritical fluid (SCF), is known to be a multipurpose and green-processing platform for the pharmaceutical industry. Basically, SCFs exhibit peculiar physical features, among which is the one that is close to the liquid one, diffusivity, and solvating power that can be adjusted (1), (2). Because of these features, they are recognized as a perfect medium for the enhancement of solubility, extraction, and particle engineering. CO_2_ SFC has been the leading choice among other SFCs due to its mild critical conditions (Tc = 304.1 K, Pc = 73.8 bar), non-toxic, non-flammable, relatively inexpensive, and easy to recover. These features have made it applicable widely in, for example, nanoparticle production, drug micronization, food extraction, and the isolation of bioactive compounds (3). In pharmaceutical science, the role of solubility of drugs in SC-CO_2_ is an important one in the decision of the processes' feasibility, such as RESS, SAS, and SSI (4, 5).

This is one of the requirements for the solubility data, which are generally taken as mandatory not only for the improvement of dissolution and bioavailability of the drugs, whose solubility in water is poor, but also for the further design of the routes of formulation, which are environmentally friendly and can be scaled up. The SC-CO_2_-based micronization methods frequently have better performances than the traditional ones (e.g., grinding, spray-drying, freeze-drying) in the aspects of narrower particle size distribution, non-occurrence of high thermal stress, and no solvent residue of a harmful nature (6, 7). However, the effectiveness and genuineness of these methods were considerably dependent on the extent to which the solubility of drugs under different temperatures and pressures was known. Equilibrium solubility in SC-CO_2_ has traditionally been determined through experiments that involve gravimetric, spectroscopic, or chromatographic methods (8, 9). Such measurements, while precise, are quite laborious, need a specially constructed high-pressure apparatus, and are usually confined to small process ranges. Consequently, there is a scarcity of experimental data, particularly for complex drug molecules, which poses a challenge in interpreting the results and further in process design (10).

### 1.2 Literature review

Sodeifian et al. (11) studied the solubility of gemcitabine, an anticancer drug, in SC-CO_2_ to support the use of supercritical fluid technology in the pharmaceutical field. The researchers measured solubility experimentally at 308–338 K and 120–270 bar by a static method with an analysis. The data showed the solubility to be extremely low (from the mole fraction) and the solubility to be of a retrograde nature in the pressure range of 190–200 bar. The goal was to capture the phenomena by fitting the data with several thermodynamic models, including both the semi-empirical and the EoS approaches. The outcome of the Peng–Robinson model was closest to the experimental data at lower temperatures, while at higher temperatures, the Soave–Redlich–Kwong model gave better results. The research work provides a way to estimate gemcitabine solubility in SC-CO_2_ and also highlights the importance of model selection for reliable pharmaceutical solubility prediction. Bahrami et al. (12) utilized ML methods to estimate the solubility of solid drugs in CO_2_. They amassed 1,816 data points from experiments, forming a comprehensive database that covers a wide range of pressures (80–400 bar), temperatures (308–348.2 K), molecular weights, and melting points of drugs. Two AI techniques were used as a basis for comparison, namely, GEP and ANFIS. The outcome revealed that ANFIS was the most accurate, showing a strong predictive performance (*R*^2^ ≈ 0.99 and RMSE ≈ 0.26) both for the training and the test sets. The analysis of the sensitivity revealed that the molecular weight of the drug was the leading factor influencing the prediction of solubility, with pressure, melting point, and temperature, in that order, following (13). This research has demonstrated the machine learning capability, especially ANFIS, as a strong alternative solution for the SC-CO_2_ solubility model.

Alsaab and Althobaiti (14) researched the estimation of phenytoin solubility in SC-CO_2_ to facilitate nanomedicine preparation and drug bioavailability improvement. The investigation utilized a dataset made up of temperature, pressure, solubility, and solvent density values. Three predictive models, ARDr, GPR, and LR, were created, with ensemble learning combined by the AdaBoost method to enhance the accuracy. The hyperparameter tuning was done with the help of JOA. The ADA-GPR ensemble outperformed other models in terms of predictive capabilities (*R*^2^ = 0.996), while ADA-LR and ADA-ARD were also close runners (*R*^2^ = 0.934 and 0.952, respectively). Wu et al. (15) examined the prediction of raloxifene solubility as well as CO_2_ density in SC-CO_2_ through the use of bagging-based regression models. They built three different models, namely: BAG-BRR, BAG-LR, and BAG-PR, and were able to tune hyperparameters using the Tree-Based PEA. Out of the three, the performance of BAG-PR was the highest, when it achieved the *R*^2^ values of 0.986 for both CO_2_ density and raloxifene solubility, accompanied by low RMSE and AARD%, which is indicative of high predictive accuracy (16). Even though the two regression methods, BAG-BRR and BAG-LR, allowed for the prediction of the target variables under study with some accuracy, their results were less precise when compared to the results of BAG-PR. The article elaborated on the potential of bagging ensemble methods with the aid of hyperparameter tuning to practically forecast pharmaceutical solubility and solvent properties in SC-CO_2_.

Makarov et al. (17) concentrated on enhancing the prediction of solubility in SC-CO_2_ with a massive databank of 31,975 records that span many chemical compounds, particularly drug-like molecules. Their research presented a machine learning model guided by thermodynamics, which allows the key features of the solute, such as enthalpy of vaporization, critical parameters, MP, and Gibbs free energy of solvation, to be merged into the predictive models. Two main approaches were utilized in this study: the CAT algorithm and a graph-based directed message-passing architecture (18). Their findings show that the use of thermodynamic descriptors from the specific domain as features in ML models leads to a big improvement in model accuracy and also its transferability to novel compounds or even mixtures.

Laggoune et al. (19) explored the solubility of sirolimus, an immunosuppressive drug, in SC-CO_2_ to facilitate pharmaceutical processing. The solubility data were obtained by a static gravimetric method. The pressure range was 12.5–25.0 MPa, and the temperature range was 313–328 K. The molar fraction varied from 1.20 × 10^−6^ to 2.73 × 10^−6^, showing a direct solubility trend within the range of parameters. For data modeling, the authors resorted to using both the density-based semi-empirical correlations and the EoS methods. Among these, the Sparks and Soave-Redlich-Kwong models gave the closest results to the experimental data, thus they were characterized by the lowest AARD% and Radj ≈ 0.978–0.980. This research presented a successful case of combining experimental measurements and calibrated thermodynamic models for the plausible prediction of sirolimus solubility in SC-CO_2_; thus, process design and optimization become easier.

Sodeifian et al. (20) explored the use of 5-F, which is an anticancer drug, in SC-CO_2_ to facilitate micro-and nanosizing processes in the pharmaceutical industry. They carried out experimental measurements at pressures of 120–270 bar and temperatures of 308–338 K with spectrophotometry. The solubility varied from 0.0024 to 0.0176 g/L, and it increased with the pressure; at constant temperatures, a crossover point was observed. The authors considered seven density-based semi-empirical models, the Peng-Robinson equation of state, and several ML methods like DT, GB, RF, and KRr to fit the data. In comparison to other models, the Sodeifian density-based correlation was the closest to the experimental results (AARD = 4.12%). Moreover, the first time the release, vaporization, and total enthalpy of the 5-Fu-SC-CO_2_ system were identified with the help of semi-empirical correlations.

Despite the substantial progress reported in recent studies, several limitations can be identified. Thermodynamic and semi-empirical models provide physically interpretable results but often require system-specific parameterization and may lack flexibility across different compounds. Conventional machine learning approaches, including ANFIS and regression-based models, have demonstrated high predictive accuracy; however, their performance is frequently dependent on large and diverse datasets, which are not always available for pharmaceutical systems. Recent ensemble and hybrid models have improved predictive capability by combining multiple learners and optimization strategies. Nevertheless, many of these approaches focus primarily on accuracy enhancement and do not explicitly address uncertainty quantification or robustness under limited and heterogeneous data conditions. Furthermore, the integration of complementary aggregation strategies within a unified framework remains relatively underexplored. In this context, the present study aims to develop a hybrid ensemble framework that integrates boosting-based learners with optimization and uncertainty-aware aggregation techniques. The objective is not to introduce a fundamentally new algorithm, but to provide a structured and robust modeling approach that improves predictive stability and reliability for solubility estimation in supercritical CO_2_ systems with limited data availability.

### 1.3 Objective and novelty of the study

**Figure 1:**
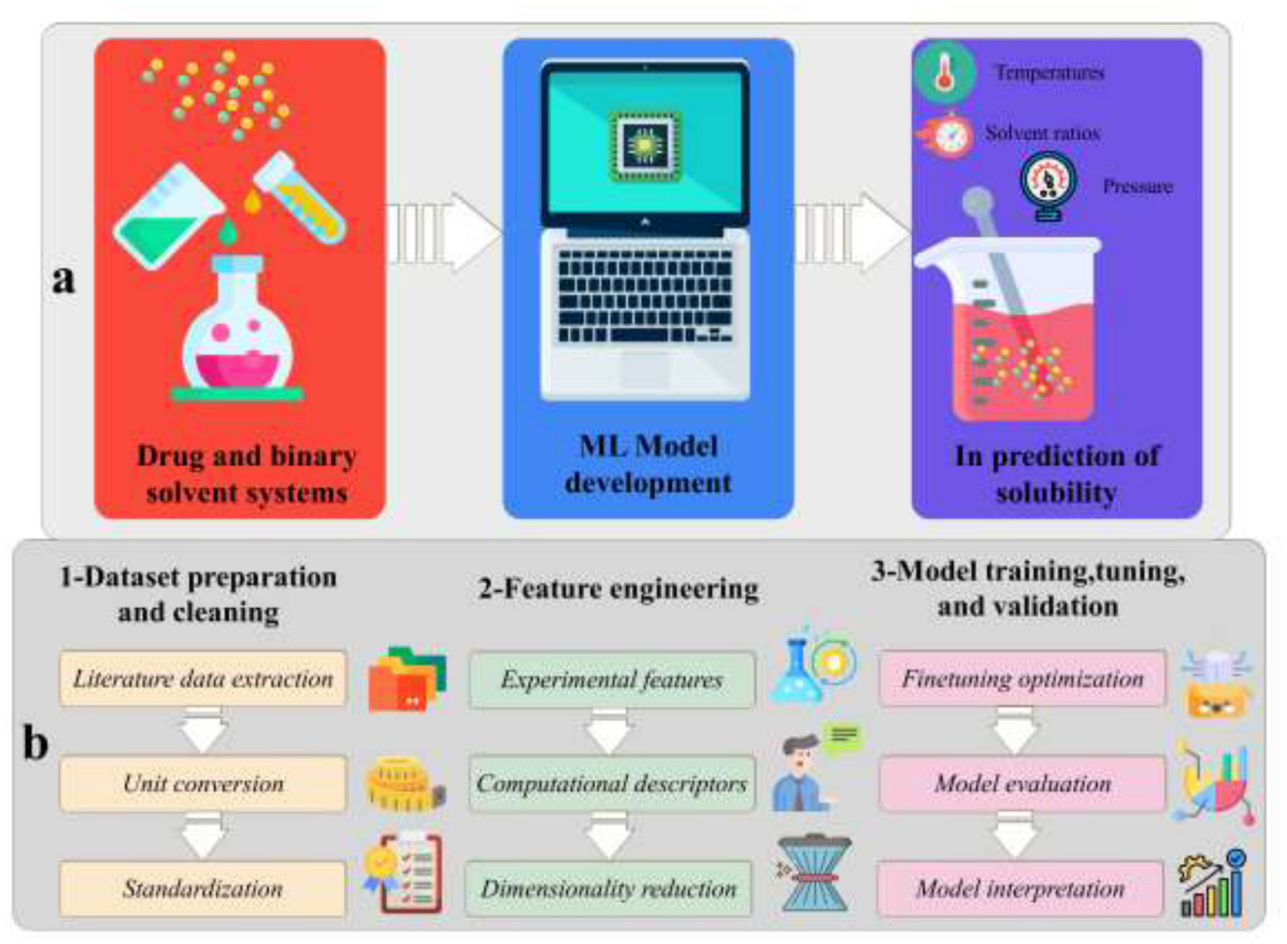
**(a)** Diagram illustrating the objective of the study, which is to predict drug solubility in supercritical CO_2_ using a machine learning-based framework. **(b)** A summary of the methods used in this investigation. After a thorough literature analysis, a complete dataset was assembled, and the data were then cleaned and standardized. The dataset was then adjusted to reduce the hazards associated with high-dimensional datasets by including both computational and experimental descriptors as input characteristics. Finally, the models' hyperparameters were optimized using this improved dataset, resulting in the selection of the best-performing models. These models were identified, confirmed experimentally, and subsequently interpreted.

The present study develops a hybrid predictive framework that integrates established machine learning techniques within a unified architecture tailored to supercritical CO_2_ solubility prediction. Rather than introducing entirely new algorithms, the approach combines boosting-based regressors with complementary ensemble strategies and optimization techniques to improve predictive stability and robustness under limited and heterogeneous data conditions. The purpose of the present study is to create a method that employs a combination of approaches and that thoughtlessly predicts the solubility of a drug in SC-CO_2_ regardless of the drug class. The idea is that different technical concepts, such as gradient boosting regressors, a DST ensemble, and hyper-parameter and feature optimization by EEFO, are united in this study. The model uses molecular descriptors, thermodynamic parameters, and process variables to represent the complex non-linear solubility behavior of different compounds; thus, it is of a high degree of transferability. The study primarily focuses on three novel aspects, namely: first, Multi-drug predictive modeling that extends beyond single-compound studies to substantiate a framework applicable to structurally diverse and poorly soluble drugs. Secondly, the Hybrid integration of domain knowledge and data-driven learning combines thermodynamic and molecular descriptors with ensemble machine learning to enhance both accuracy and interpretability. Thirdly, Bio-inspired optimization for ensemble tuning, represented by the EEFO algorithm, would be beneficial in enhancing feature selection and model stability, thereby establishing it as the primary method for SC-CO_2_ solubility prediction. Figure 1 shows the scheme of the objective of the study and the methodology used.

Although the dataset comprises a limited number of compounds, the generalization capability of the proposed framework is supported by the structural diversity of the selected drugs and the hybrid representation of input features. The investigated compounds span a wide range of molecular weights, melting points, and solubility regimes, enabling the model to capture generalized physicochemical relationships rather than compound-specific trends. In addition, the integration of thermodynamic variables (temperature and pressure) with molecular descriptors provides a unified feature space that enhances model transferability across different drug classes under supercritical CO_2_ conditions. The use of ensemble learning and EEFO-based optimization further improves robustness by reducing overfitting and stabilizing predictions under limited data availability. To mitigate potential overfitting, multiple validation strategies, including cross-validation, independent testing, and uncertainty quantification, were employed. It is acknowledged, however, that the applicability of the model is constrained to compounds and operating conditions within the range of the training data. Therefore, the framework is best interpreted as a transferable predictive tool within similar thermodynamic and molecular domains, rather than a universally generalizable model.

## 2 Materials and methods

### 2.1 Data description

The dataset that was utilized in the research paper were gathered from four separate experimental studies (19, 21–23) on the solubility of drugs in SC-CO_2_. Their combined studies provided 110 solubility measurements at equilibrium made under controlled high-pressure and high-temperature conditions using the standard SC-CO_2_ static equilibrium methods. The range of experimental conditions covered was with pressures from 12 to 30 MPa and temperatures from 308 to 338 K (some studies were with co-solvent ethanol at 3 mol%). The choice of these four compounds was based on the fact that they are common water-insoluble drugs of pharmacological significance, thereby constituting four structurally radically different drug classes (antibiotics, immunosuppressants, anticancer agents). For the modeling purpose, the data set had been designed with four input features and one output variable:

- Temperature (K) affected the density of the solvent and the solvation capacity.
- Pressure (MPa) was the main factor that changed the phase of CO_2_, its density, and these were the direct changes that had an impact on the solubility.
- Molecular weight (g/mol) was the property that represented diffusivity limitations and steric effects.
- Melting point (°C) showed the crystalline stability and sublimation tendency.
- Output variable: solubility (g/L × 10) was the equilibrium solubility in SC-CO_2_ that was measured experimentally.

Table 1 provides a summary of the experimental ranges for each compound, accompanied by their physicochemical descriptors (molecular weight and melting point). The data reveal notable variations among the drugs: rifampin and teriflunomide are, for example, the two compounds with the widest solubility ranges, whereas Sirolimus has a limited solubility window even though its molecular weight is high. Such differences highlight the range of structures and energetics in the dataset, which in turn, represents a challenging test for the predictive models to be developed.

**Table 1:** Overview of input features and output variables with their statistical properties.

Drug	Chemical formula	Features	Unit	Statistical properties			
				Min	Max	Average	St. dev
Tacrolimus	C_44_H_69_NO_12_	Temp	(k)	308	338	323	11.1803
		Pressure	(Mpa)	12	30	21	6
		Molecular weight	(g/mol)	—	—	804.03	—
		Melting point	(C)	—	—	128	—
		Solubility	(g/L) ^*^ 10	0.029	0.235	0.1375	0.0550
Rifampin	C_43_H_58_N_4_O_12_	Temp	(k)	308	338	323	11.1803
		Pressure	(Mpa)	12	30	21	6
		Molecular weight	(g/mol)	—	—	822.95	—
		Melting point	(C)	—	—	185	—
		Solubility	(g/L) ^*^ 10	0.109	2.983	1.73396	0.7639
Teriflunomide	C_12_*H*_9_F_3_N_2_O_2_	Temp	(k)	308	338	323	11.1803
		Pressure	(Mpa)	12	30	21	6
		Molecular weight	(g/mol)	—	—	270.21	—
		Melting point	(C)	—	—	230	—
		Solubility	(g/L) ^*^ 10	0.127	3.212	1.4381	0.7810
Sirolimus	C_51_H_79_NO_13_	Temp	(k)	313	328	321.4211	5.6318
		Pressure	(Mpa)	12.5	25	19.4736	3.8541
		Molecular weight	(g/mol)	—	—	914.172	—
		Melting point	(C)	—	—	183	—
		Solubility	(g/L) ^*^ 10	0.2122	0.4567	0.3173	0.0671

In the present framework, supercritical CO_2_ density was not explicitly included as an input variable, as it is a thermodynamic property determined by temperature and pressure through the equation of state. The model therefore relies on temperature and pressure as primary descriptors of the system state to avoid redundancy in the feature space. Nevertheless, it is recognized that the explicit incorporation of density as an input feature may enhance the physical interpretability and predictive capability of the model. Previous studies have demonstrated that density can serve as a key variable governing solubility behavior in supercritical systems. Accordingly, future work will investigate the integration of density and other thermodynamic descriptors to further improve model performance and physical consistency.

**Figure 2:**
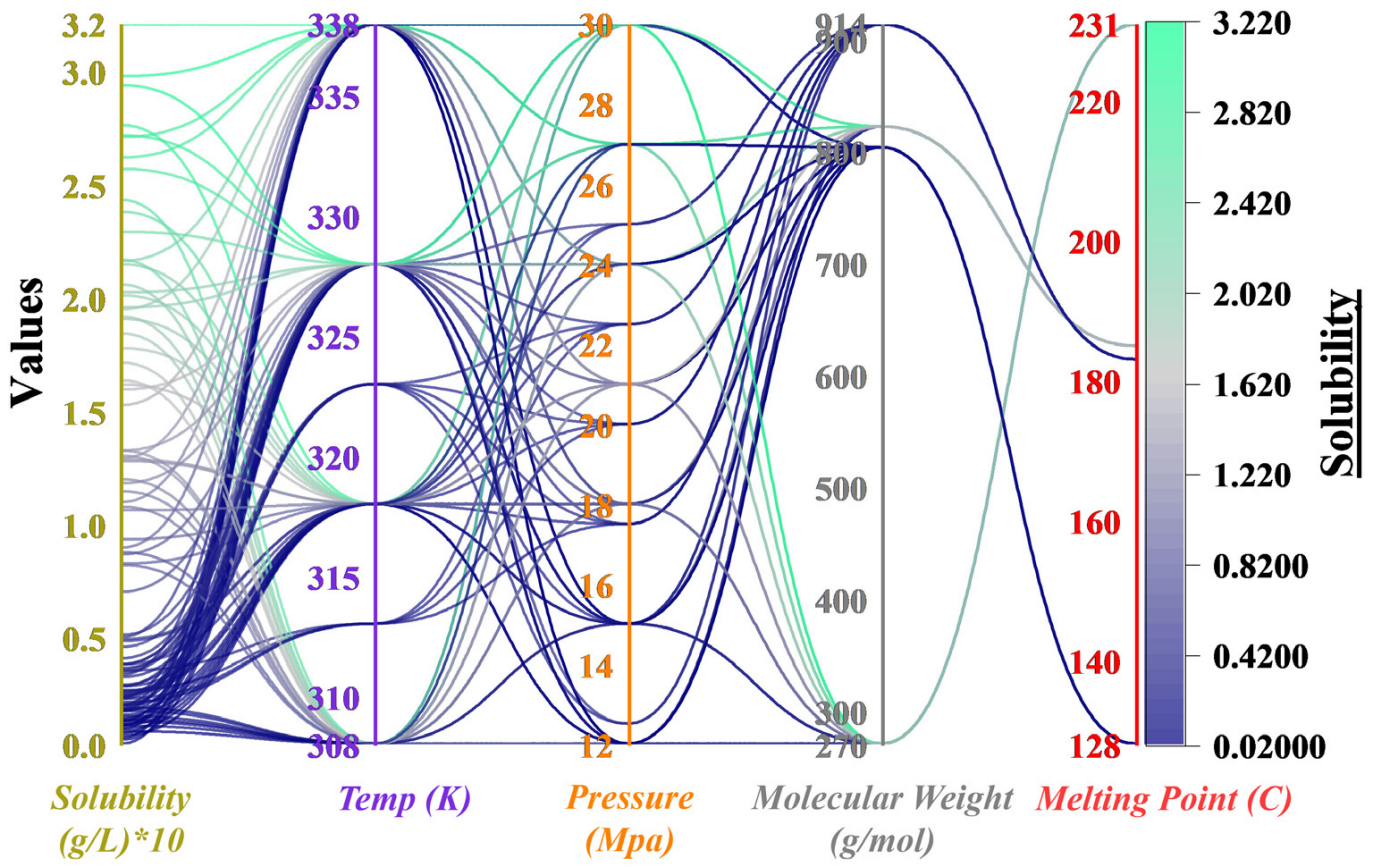
Parallel coordinates plot of experimental solubility data in SC-CO_2_, with solubility mapped as a color gradient. The plot illustrates the multivariate relationships among solubility, temperature, pressure, molecular weight, and melting point for the four investigated drugs.

Figure 2 indicates a Global Visualization of the Dataset with a parallel coordinates framework, in which each polyline represents one experimental observation. Solubility, for example, is positively associated with both temperature and pressure, which aligns with the physical changes of SC-CO_2_ vapor and density pressure that facilitate the dissolution process. Nevertheless, it is not a strict linear relation; Rifampin shows a non-monotonic profile under some pressure–temperature combinations. Besides, molecular weight and melting point have opposite effects on each other: the four compounds with the highest molecular weights (Sirolimus, Tacrolimus) and the lowest melting points are those that, most likely, will have the highest solubilities; at the same time the ones with lower molecular weights and higher melting points (e.g., Teriflunomide) will have lower solubility values.

**Figure 3:**
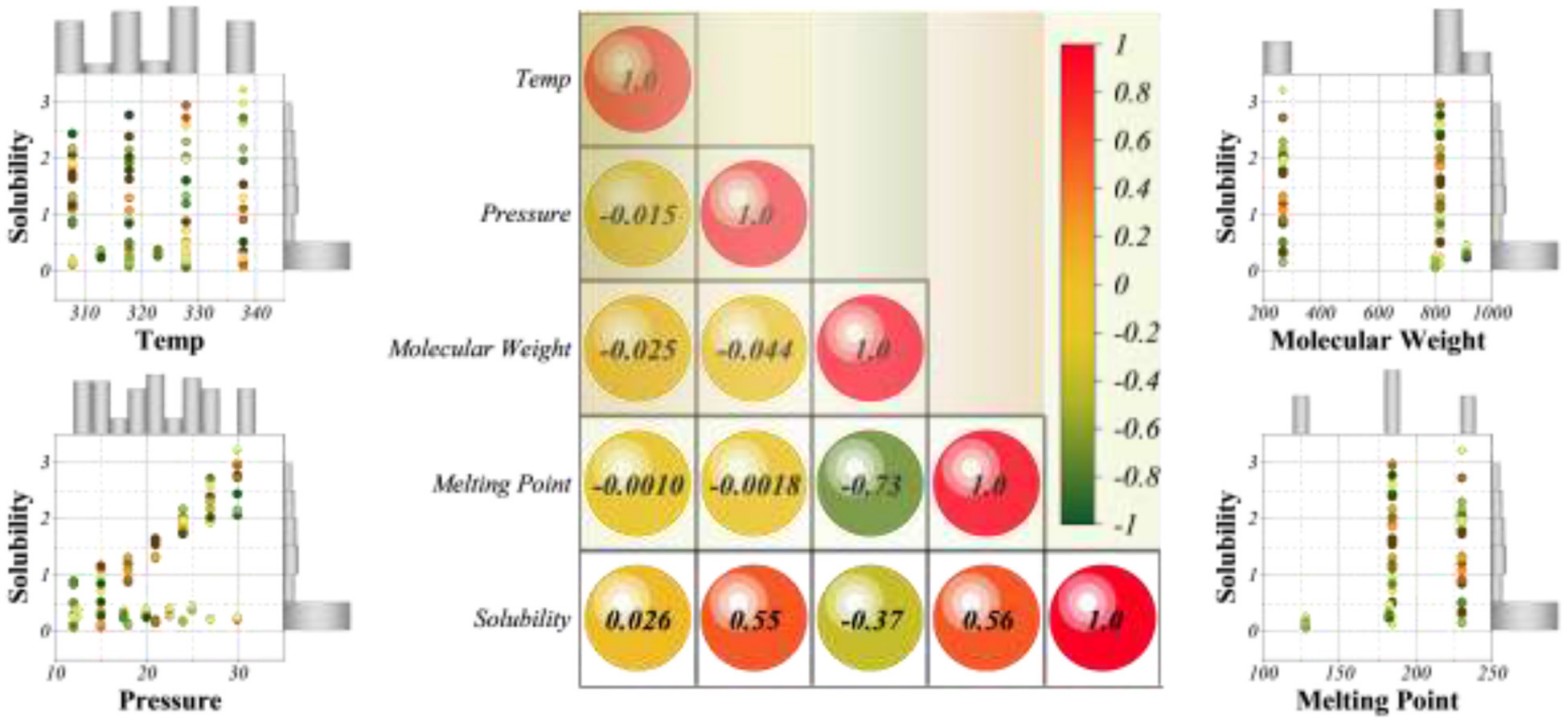
Correlation matrix and pairwise scatter plots of the input features and solubility in SC-CO_2_. The central heatmap shows Pearson correlation coefficients, while the scatter plots and histograms illustrate bivariate and univariate distributions, respectively.

Figure 3 presents the correlation structure of the dataset. The Pearson coefficients indicate that solubility has a moderate positive correlation with pressure (0.55) and melting point (0.56), while its correlation with molecular weight is moderately negative (−0.37). Temperature shows only a weak correlation (0.026), suggesting that its effect is non-linear and intertwined with pressure, consistent with supercritical fluid behavior. Among the input features, melting point and molecular weight exhibit a strong negative correlation (−0.73), which reflects the physicochemical trade-off between crystalline stability and molecular size across the four studied drugs. The scatter plots confirm these trends: solubility increases with pressure in a quasi-linear fashion, while molecular weight introduces dispersion, with heavier compounds showing consistently lower solubility values.

### 2.2 Base learners

#### 2.2.1 Gradient Boosting Regression (GBR)

A strong predictive model in boosting is created through the iterative refinement of weak learners, where each new learner is trained to address the mistakes made in earlier stages. Think of a set of random response variables, and a collection of random input variables. The goal is to determine the function *F*(*x*) approximation $\tilde{F}\left(x\right)$ mapping *x to y* to lower the loss function *L*(*y, F* (*x*)) utilizing training dataset in the form of {(*x*_*i*_, *y*_*i*_)}. Making errors is a given (Equation 1) while trying to discover the formula *F*(*x*).


$$\begin{array}{c} \tilde{F}\left(x\right)=arg\;\underset{F(x)}{\min}L_{y,x}(y,F(x) \end{array}$$ (1)

To predict the approximation formula, the squared error function is used as the loss function. The GB method beginss by establishing a first base learner *F*_0_(x) (step 1). The following formula (Equation 2) (step 3) may be utilized to get the the loss function's gradient *L*(*y, F* (*x*) ):


$$\begin{array}{c} \tilde{y_{i}}=-{\left[\frac{\partial L(y_{i},F(x_{i})}{\partial F\left(x_{i}\right)}\right]}_{F\left(x\right)=F_{m-1}\left(x\right)}\;i=1,\ldots ,N \end{array}$$ (2)

For the gradient, regression trees *h*(*x*_*i*_; *a*) with parameter *a* can be used as *wl* to expand the range of calculations. Usually it is a function of parameteriaing, where the input components (*x*) are represented by parameters (24). To create the tree, step 4 requires the solution of the following equation (Equation 3):


$$\begin{array}{c} a_{m}=arg\;\underset{\alpha ,\beta }{\min}\overset{N}{\underset{i=1}{\sum }}{\left[\tilde{y_{i}}-\beta h\left(x_{i},\alpha \right)\right]}^{2} \end{array}$$ (3)

**Figure 4:**
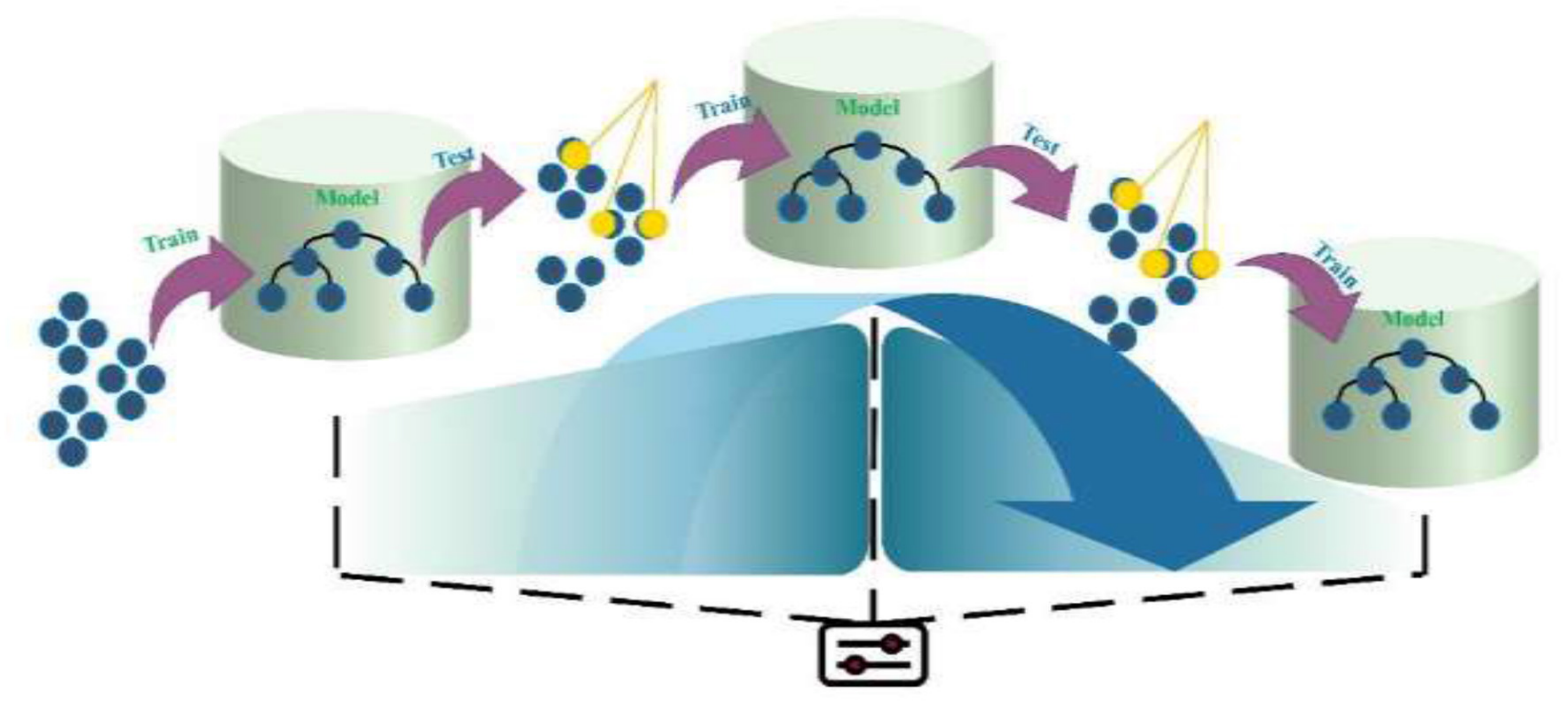
The structure of the GBR model.

The set of parameters learned at iteration m is denoted by *a*_*m*_, with β representing the weight value, commonly referred to as the expansion coefficient, assigned to each *wl*. The negative gradient used to fit every regression tree. After that, step 5 finds the optimum length *m*, and step 6 updates the model *F*_*m*_(*x*) for each iteration m. Figure 4 shows the structure of the GBR model.

#### 2.2.2 Histogram Gradient Boosting Regression (HGBR)

The HGBR is based on collaborative decision trees. In order to improve performance, the method greedily adds additional corrective models incrementally, decreasing the loss's squared error function until it is acceptable. The histogram is a useful data structure that is used in the tree-building approach to expedite the process.

**Figure 5:**
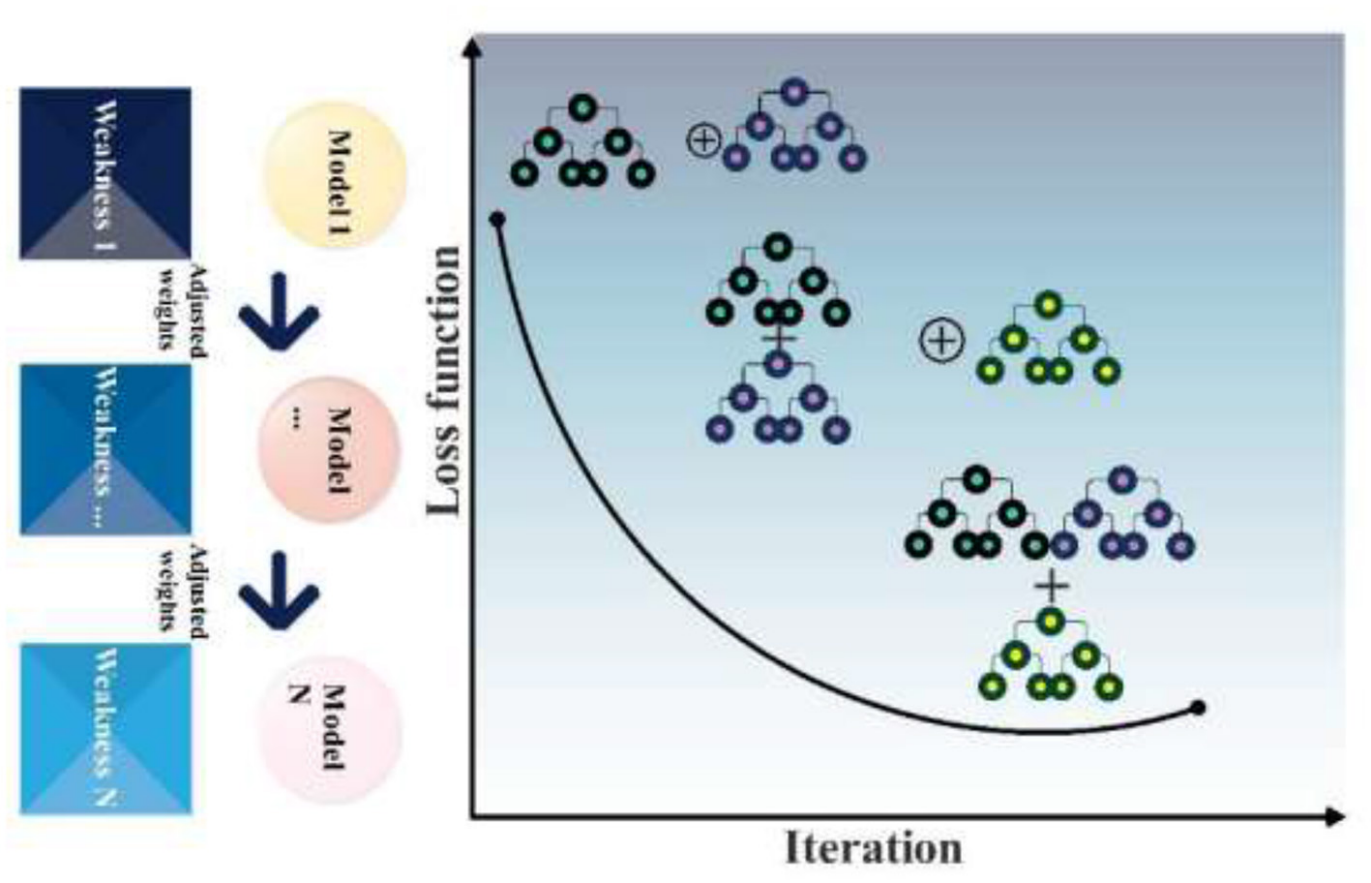
The structure of the HGBR model.

The HGBR technique uses gradient boosting to speed up decision tree training. The training of recently added trees in an ensemble can be significantly accelerated by discretizing or binning. The HGB method then executes its algorithm using the input variables. Each tree added to an ensemble aims to address anticipated issues by leveraging the models already present. The HGBR methodology is used in addition to other procedures. Scikit-learn ML, a toolkit offering GB experimentaly implementation, may be utilized to execute the procedure. These classes are the HGBR and the Gradient Boosting. The scikit-learn records show that the HGBR implementation is significantly faster than the library's default GBR version (25, 26). Figure 5 shows the structure of the HGBR model.

#### 2.2.3 Rationale for model selection and hyperparameter tuning

The choice of GBR and HGBR as base learners was motivated by their strong ability to capture complex non-linear dependencies between input features and solubility outcomes. Unlike linear or shallow learners, boosting-based algorithms construct ensembles of weak learners that iteratively correct prediction errors, thereby offering improved accuracy and generalization. These features make them stable when dealing with small and diverse datasets, which are typical in pharmaceutical and supercritical fluid solubility research. In addition, both GBR and HGBR have been cited many times as the main sources of trustworthy predictions in chemical engineering and pharmaceutical modeling tasks, which is a strong argument for their use in the current framework. Essential hyperparameters of the models were systematically varied to increase the prediction quality and security. Namely, the learning rate was set with the aim of achieving a proper trade-off between the speed of convergence and the avoidance of overfitting, the maximum tree depth was adjusted to limit model complexity, and the degree of feature interactions was adjusted. Finally, the number of boosting stages was selected in such a way that the ensemble had room for further training but without the risk of overfitting. These changes in hyperparameters, excluding the EEFO algorithm, allowed for an efficient search across the space of parameters, thus enabling the base learners to perform their best under such conditions for drug solubility in SC-CO_2_.

### 2.3 Ensemble framework

#### 2.3.1 Dempster–Shafer theory (DST) ensemble

The DST, presented initially by Dempster in 1967 (27) and subsequently developed by Shafer in 1976 (28), is an extension of Bayesian probability theory that aims at handling uncertainty and partial knowledge explicitly. It differs from classical probability, which necessitates the provision of exact probabilities for mutually exclusive events, in that it permits the allocation of belief to sets of hypotheses. The framework is particularly well-suited for uncertain and imprecise situations observed in the engineering field, such as when variability in the model or heterogeneously structured data is involved (29), (30). DST is a theory of evidence that assigns degrees of belief based on the evidence at hand. It works mainly through two main features: the belief function that quantifies the minimum amount of support, which the evidence provides to the hypothesis, and the plausibility function, which represents the maximum level of support not opposed by the evidence. The difference between the two functions gives the “ignorance interval,” which represents the uncertainty or lack of knowledge due to the proposition. Therefore, DST is a more suitable method for complex and uncertain prediction cases.

#### 2.3.2 Weighted average ensemble

Besides the Dempster-Shafer theory-based fusion, a weighted averaging scheme was also implemented as an alternative ensemble strategy. The weighted ensemble method merges the predictions of several base learners by giving each model output a weight that has been optimized, rather than the resulting prediction being treated as a simple average. This feature enables the final output to be strengthened by leveraging the shared advantages of the different models, while simultaneously mitigating their weaknesses. In the current study, GBR and HGBR were identified as the primary learners of the core because of their demonstrated ability to manage non-linear relationships and scarce experimental data successfully.

The reasons for the use of a weighted average ensemble lie in the fact that this method is simple, easy to understand, and robust. The ensemble, by varying the amount of each model according to its predictive accuracy, results in a more stable and generalized outcome than that of any single learner. The EEFO algorithm was the means by which the weight was optimized. It effectively searched for the best weight setup by balancing exploration and exploitation of the solution space. As a result, the final weighted model was a reflection of the bias–variance trade-off that was specifically targeted at the solubility prediction task. The weighted averaging ensemble not only provided increased reliability of the predictions but also opened up the possibility of assessing the relative importance of individual base learners in an interpretable way, thus offering a viable and computationally efficient approach for modeling drug solubility in supercritical CO_2_.

#### 2.3.3 Ensemble integration strategy

To further enhance predictive robustness, the proposed framework integrates multiple learners using two complementary ensemble strategies: weighted averaging and Dempster–Shafer theory (DST) fusion. In the weighted averaging approach, individual model predictions are linearly combined according to optimized weights, which are determined through the Electric Eel Foraging Optimization (EEFO) algorithm. This ensures that base learners with higher predictive accuracy contribute more strongly to the outcome, while weaker learners exert proportionally less influence.

In contrast, the DST-based fusion accounts for uncertainty and partial evidence by assigning belief and plausibility values to the outputs of each model. This probabilistic reasoning mechanism enables a more flexible integration when predictions diverge, particularly under conditions of data heterogeneity or limited experimental observations. The combination of these ensemble techniques was specifically chosen to address two critical challenges: (i) variance reduction, by stabilizing fluctuations arising from small-sample datasets and non-linear response surfaces, and (ii) improved generalization, by leveraging the complementary error patterns of different base learners. In general, these strategies yielded a hybrid ensemble that is both computationally efficient and capable of producing more reliable solubility predictions across structurally diverse pharmaceutical compounds in supercritical CO_2_.

Unlike conventional ensemble learning approaches that primarily rely on simple averaging or stacking techniques, the proposed aggregation strategy integrates both deterministic and uncertainty-aware fusion mechanisms. The weighted averaging component provides an optimized linear combination of base learners, where model contributions are adaptively adjusted based on their predictive performance through the EEFO algorithm. In parallel, the Dempster–Shafer theory (DST)-based fusion introduces an additional layer of probabilistic reasoning by explicitly accounting for uncertainty in model predictions. Through the assignment of belief and plausibility measures, DST enables flexible aggregation when individual model outputs are inconsistent or partially conflicting. The combination of these two complementary strategies distinguishes the proposed framework from conventional ensembles by not only improving predictive accuracy but also enhancing reliability under conditions of limited and heterogeneous data. This hybrid aggregation mechanism allows the model to balance performance and uncertainty, thereby providing a more robust predictive tool for solubility estimation in supercritical CO_2_ systems.

### 2.4 Optimization with electric eel foraging

#### 2.4.1 EEFO

Establishing basic parameters control, such as the max iteration count and the electric eel population size, is the preliminary phase of the EEFO technique (31). When the energy factor (*E*) is *one* or less, each eel participates in exploitation with equal likelihood. The randomly generated eels demonstrate exploratory behavior when their energy factor (*E*) exceeds *one*. The process generates candidate solutions, compares them with the current solution, and iteratively updates the best option. As the iteration time grows, the energy factor (*E*) falls, suggesting a shift from exploration to exploitation. It can handle a broad variety of optimization problems, including ones with many variables and constraints. The EEFO takes into consideration unethical elements of problem-solving (Equation 4).


$$\begin{array}{c} X_{i}\left(t+1\right)=\{\begin{array}{c} X_{i}\left(t\right)\;fit\left(X_{i}\left(t\right)\right)\leq fit\left(v_{i}\left(t+1\right)\right)\\ v_{i}\left(t+1\right)\;fitfit\left(X_{i}\left(t\right)\right)>fit\left(v_{i}\left(t+1\right)\right) \end{array} \end{array}$$ (4)

Here, *X*_*i*_ indicates the an eel randomly selected from the current population position, *v*_*i*_ indicates the location of a randomly picked meal, and *fit*(*X*_*i*_(*t*)) indicates the fitness of the proposed position of the *i*-th electric eel. Setting control parameters, such as the max iterations number and the population size of the *ee*. Next, a random collection of eels is generated and distributed. Whenever the *E* is larger than one, each eel employs its interactive behavior to explore throughout each cycle. Conversely, every eel has the same chance of participating in exploitative behavior when the energy component *E* is one or less. Each example is run through each eel in order to develop new potential solutions, compared to the ones that currently exist. Throughout the iteration process, the best solution is updated continuously. Every eel moves from exploration to exploitation when the energy factor *E* reduces over the course of the iteration. Until the designated stop condition is satisfied, this interactive process keeps going (32).

#### 2.4.2 Optimization strategy with EEFO

The optimization phase in this study targeted two main components: (i) the hyperparameters of the base learners, including learning rate, maximum depth of trees, and number of boosting iterations, and (ii) the ensemble weights governing the contribution of individual models within the hybrid framework. Proper calibration of these parameters was essential to maximize predictive accuracy while ensuring robustness and transferability across structurally diverse drugs.

The EEFO algorithm was employed as the optimization engine due to its adaptive balance between exploration and exploitation. In this study, the EEFO was initialized with a population size of 30 candidate solutions and run for a maximum of 200 iterations, with convergence determined when no improvement in fitness was observed for 20 consecutive iterations. The algorithm dynamically adjusted the energy factor of each candidate to transition from global exploration in the early stages to local exploitation in later stages, thereby ensuring efficient search across the parameter space. The choice of EEFO over conventional optimization algorithms (random search) was justified by three key advantages: (i) its ability to capture global optima in highly non-linear and multi-dimensional search spaces, (ii) its inherent mechanism to avoid premature convergence and overfitting, and (iii) its proven stability across diverse engineering and chemical modeling tasks. By leveraging these features, the EEFO-based optimization significantly improved both the accuracy and generalization of the ensemble models for solubility estimation in SC-CO_2_.

The selection of the EEFO algorithm was guided by its capability to effectively explore complex and non-linear optimization landscapes associated with ensemble learning models. Unlike conventional approaches such as grid search and random search, which require extensive evaluations and may fail to capture global optima, EEFO employs a population-based search strategy that dynamically balances exploration and exploitation. In comparison to more established techniques such as Bayesian optimization, EEFO does not rely on surrogate modeling or prior assumptions about the objective function, making it more flexible in handling irregular and multi-modal search spaces. This characteristic is particularly relevant for the present framework, where interactions between model hyperparameters and ensemble weights introduce additional complexity. Furthermore, the adaptive energy-based mechanism in EEFO reduces the risk of premature convergence and improves stability across iterations, as reflected in the convergence behavior presented in Section 3.1. Therefore, EEFO is adopted as an efficient and robust optimization tool tailored to the requirements of solubility prediction in supercritical CO_2_ systems, rather than as a replacement for established optimization methods.

### 2.5 Model validation and statistical analysis

#### 2.5.1 Cross-validation

The predictive robustness of the developed models was assessed through a 5-fold cross-validation process, which is widely regarded as a rigorous approach for evaluating model generalizability on limited datasets. The data was randomly divided into five mutually exclusive subsets (K1–K5). For each iteration, four fold were employed for training, and the remaining fold was used for validation, such that each subset contributed once to the validation process. This systematic resampling approach reduces partition bias and provides a more reliable estimate of model performance compared to a single hold-out method. In addition to cross-validation, an independent test set was defined and excluded from the training and hyperparameter optimization processes. This test subset was used exclusively for the final evaluation of model performance, ensuring that predictive accuracy reflects true generalization to unseen data rather than resampling-based estimates. Accordingly, performance metrics reported in the Results section are presented separately for training (cross-validation) and testing phases to provide a clear distinction between model fitting and independent validation.

**Figure 6:**
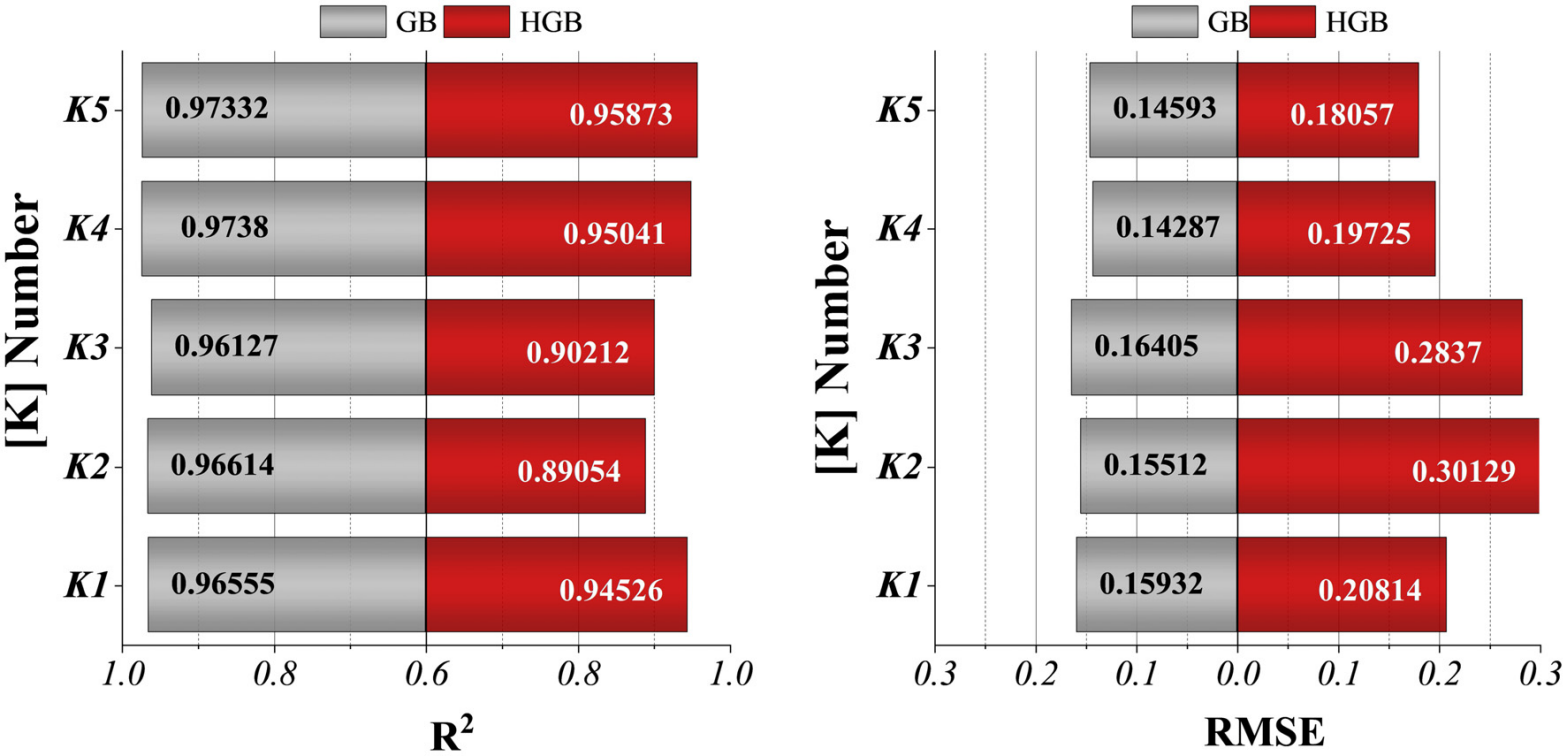
Population pyramid plot for visualizing the distribution of samples across folds in K-fold cross-validation.

The cross-validation outcomes, summarized in Figure 6, were quantified using the R^2^ and the RMSE. Across all folds, both GB and HGB yielded high R^2^ values (0.89–0.97), indicating a strong capacity to explain the variance in drug solubility data. Correspondingly, RMSE values remained low and within a narrow range (0.14–0.30), which demonstrates the stability of predictive accuracy. A comparative analysis between the two learners revealed that GB consistently achieved marginally superior performance, with higher R^2^ and lower RMSE across most folds. This suggests that GB possesses enhanced robustness in modeling non-linear solubility behavior under the constraints of relatively small experimental datasets. Collectively, the cross-validation results validate the reliability of the boosting-based learners and justify their subsequent integration into hybrid and ensemble frameworks.

#### 2.5.2 Statistical significance

The statistical relevance of the input features is examined using the Kruskal–Wallis test, a non-parametric method that evaluates whether solubility distributions vary significantly across feature-defined groups. This approach is particularly appropriate for non-linear and non-normally distributed data, such as those encountered in supercritical solubility studies. The test highlights that melting point and molecular weight contribute the most significant variation in solubility, followed by pressure, whereas temperature shows relatively weaker discriminatory power. These findings emphasize the dominant role of structural and thermodynamic descriptors over modest thermal effects in determining solubility behavior.

#### 2.5.3 Uncertainty estimation

Uncertainty estimation was conducted using a bootstrapped prediction interval (PI) approach, in which the dataset was resampled with replacement to generate multiple pseudo-training sets, and the model was retrained on each replicate to produce a distribution of predictions. The 95% prediction intervals were then derived from the percentile bounds (2.5th and 97.5th percentiles) of these distributions. In predictive modeling, particularly in pharmaceutical and supercritical fluid applications, quantifying uncertainty is as critical as reporting central accuracy metrics such as RMSE or *R*^2^. Since the experimental dataset is relatively small and heterogeneous, there exists an inherent risk of overfitting or model miscalibration when applied to unseen drug compounds or new operating conditions. To address this, uncertainty estimation was incorporated into the modeling framework using a prediction interval bootstrapping approach.

Bootstrapping was conducted by resampling the dataset with replacement to generate multiple pseudo-training sets of the same size as the original. For each bootstrap replicate, the hybrid ensemble model (GB–HGB–EEFO) was retrained and used to predict the solubility outcomes of the validation samples. Repeating this procedure for 1,000 iterations produced an empirical distribution of predictions for each observation, from which prediction intervals (PI) were derived. Specifically, the lower and upper bounds of the PI were obtained by computing the 2.5th and 97.5th percentiles of the bootstrapped predictions, corresponding to a 95% confidence level.

The interpretation of these intervals provides two fold insights. First, the width of the PI reflects the degree of uncertainty associated with a particular prediction: narrow intervals indicate high model confidence, whereas wider intervals denote regions of greater variability, often corresponding to sparse or structurally complex areas of the dataset. Second, the coverage probability, defined as the proportion of observed experimental solubility values that fall within their respective intervals, was evaluated as a measure of calibration. A well-calibrated model should ideally achieve coverage close to the nominal 95% level, thus ensuring that the ensemble framework does not underestimate predictive uncertainty.

By explicitly quantifying predictive reliability, bootstrapped prediction intervals enhance the interpretability and applicability of the proposed computational framework. This is particularly advantageous in pharmaceutical process design, where conservative decision-making is often required to mitigate risks associated with scale-up or regulatory compliance.

#### 2.5.4 Performance metrics

To ensure a comprehensive evaluation of the predictive framework, several complementary statistical indicators were employed. These metrics jointly assess both the accuracy of point predictions and the reliability of the associated uncertainty estimates.

##### 2.5.4.1 Root Mean Squared Error (RMSE)

RMSE penalizes larger errors more heavily, thereby reflecting the sensitivity of the model to extreme deviations. A lower RMSE indicates superior predictive performance and closer alignment with experimental data (Equation 5).


$$\begin{array}{c} RMSE=\sqrt{\frac{1}{n}\sum_{i=1}^{n}{\left(y_{i}-{\hat{y}}_{i}\right)}^{2}} \end{array}$$ (5)

##### 2.5.4.2 Coefficient of determination (R^2^)

It ranges from 0 to 1, with higher values signifying a stronger explanatory power. *R*^2^ quantifies the proportion of variance in the observed dataset that the model explains (Equation 6).


$$\begin{array}{c} R^{2}=1-\frac{\sum_{i=1}^{n}{\left(y_{i}-{\hat{y}}_{i}\right)}^{2}}{\sum_{i=1}^{n}{\left(y_{i}-\bar{y}\right)}^{2}} \end{array}$$ (6)

where $\bar{y}$ indicates the observed solubility values mean.

##### 2.5.4.3 Mean Absolute Percentage Error (MAPE)

MAPE indicates the estimation error as a percentage relative to observed values, providing an intuitive measure of average deviation across the dataset (Equation 7).


$$\begin{array}{c} MAPE=\frac{100}{n}\sum_{i=1}^{n}\left|\frac{y_{i}-{\hat{y}}_{i}}{y_{i}}\right| \end{array}$$ (7)

##### 2.5.4.4 Prediction interval coverage probability (PI)

PI coverage probability evaluates the reliability of model uncertainty quantification. It represents the fraction of experimental solubility values that fall within the model's bootstrapped prediction intervals (Equation 8).


$$\begin{array}{c} PI=\frac{1}{n}\sum_{i=1}^{n}𝕀\;\left(y_{i}\in \left[L_{i},\;U_{i}\right]\right) \end{array}$$ (8)

where *L*_*i*_ and *U*_*i*_ are the lower and upper bounds of the prediction interval for the *ith* observation, and *I* (·) is the indicator function, equal to 1 if the condition holds and 0 otherwise.

##### 2.5.4.5 Fraction of Guest Error (FGE)

FGE provides a normalized measure of predictive error relative to the observed values. It complements RMSE by assessing the proportional deviation of predictions across the dataset (Equation 9).


$$\begin{array}{c} FGE=\frac{1}{n}\overset{n}{\underset{i=1}{\sum }}\frac{\left|y_{i}-{\hat{y}}_{i}\right|}{\left|y_{i}\right|} \end{array}$$ (9)

Smaller FGE values indicate that the ensemble model consistently maintains accuracy across structurally diverse drug classes.

### 2.6 Implementation details

The computational framework developed in this study was implemented in Python 3.10, leveraging a combination of open-source machine learning libraries and a custom-coded optimization module. The scikit-learn (v1.3.2) library served as the primary platform for constructing and training the GBR and HGBR models. Data preprocessing, statistical analysis, and visualization were conducted using NumPy (v1.25.0), pandas (v2.0.3), SciPy (v1.11.1), and Matplotlib/Seaborn (v3.7.2/v0.12.2), respectively. Correlation analysis and feature importance were supplemented with LIME (v0.2.0.1) to provide interpretable insights into model predictions.

A custom implementation of the EEFO algorithm was developed in Python, following the algorithmic formulations described in Section 2.4. This module was designed to be fully modular, enabling seamless integration with the base learners and ensemble strategies. EEFO handled hyperparameter tuning (tree depth learning rate, and number of estimators) and weight optimization within the ensemble framework, with convergence criteria and fitness evaluation coded specifically for the solubility prediction task. The ensemble integration strategies were implemented through tailored functions combining weighted averaging and Dempster–Shafer Theory (DST). This dual-layer ensemble structure was constructed to maximize predictive robustness under limited and heterogeneous datasets, while ensuring that both epistemic (model-driven) and aleatoric (data-driven) uncertainties were explicitly accounted for.

All experiments were conducted in a controlled computational environment. The system was equipped with an Intel^®^ Core™ i7-12700K processor (12 cores, 3.6 GHz base frequency), 32 GB DDR4 RAM, and Windows 11 Pro (64-bit) operating system. Parallelized computation was enabled through joblib to accelerate cross-validation and bootstrapping procedures. The total runtime for model training and optimization across all bootstrap iterations was approximately 2.5 hours, underscoring the computational efficiency of the proposed hybrid ensemble approach compared to conventional thermodynamic modeling or extensive experimental measurements. By combining robust open-source libraries with custom-coded modules and executing them in a high-performance computing environment, the proposed framework balances reproducibility, scalability, and domain-specific adaptability, thereby ensuring that the methodology can be readily extended to other drug classes and supercritical fluid systems.

The computational cost of the proposed framework is inherently higher than that of individual machine learning models due to the combined effects of ensemble integration, EEFO-based hyperparameter optimization, and bootstrapped uncertainty estimation. Nevertheless, the total runtime remained within practical limits (approximately 2.5 hours on a standard workstation), making the approach suitable for offline analysis and process design applications. Importantly, once the model is trained, prediction of new samples is computationally inexpensive and can be performed in real time. Therefore, the increased training cost is offset by improved predictive accuracy, robustness, and uncertainty quantification, providing a favorable trade-off compared to simpler models in applications involving complex and limited datasets.

## 3 Result and discussion

### 3.1 Optimization and convergence behavior

**Figure 7:**
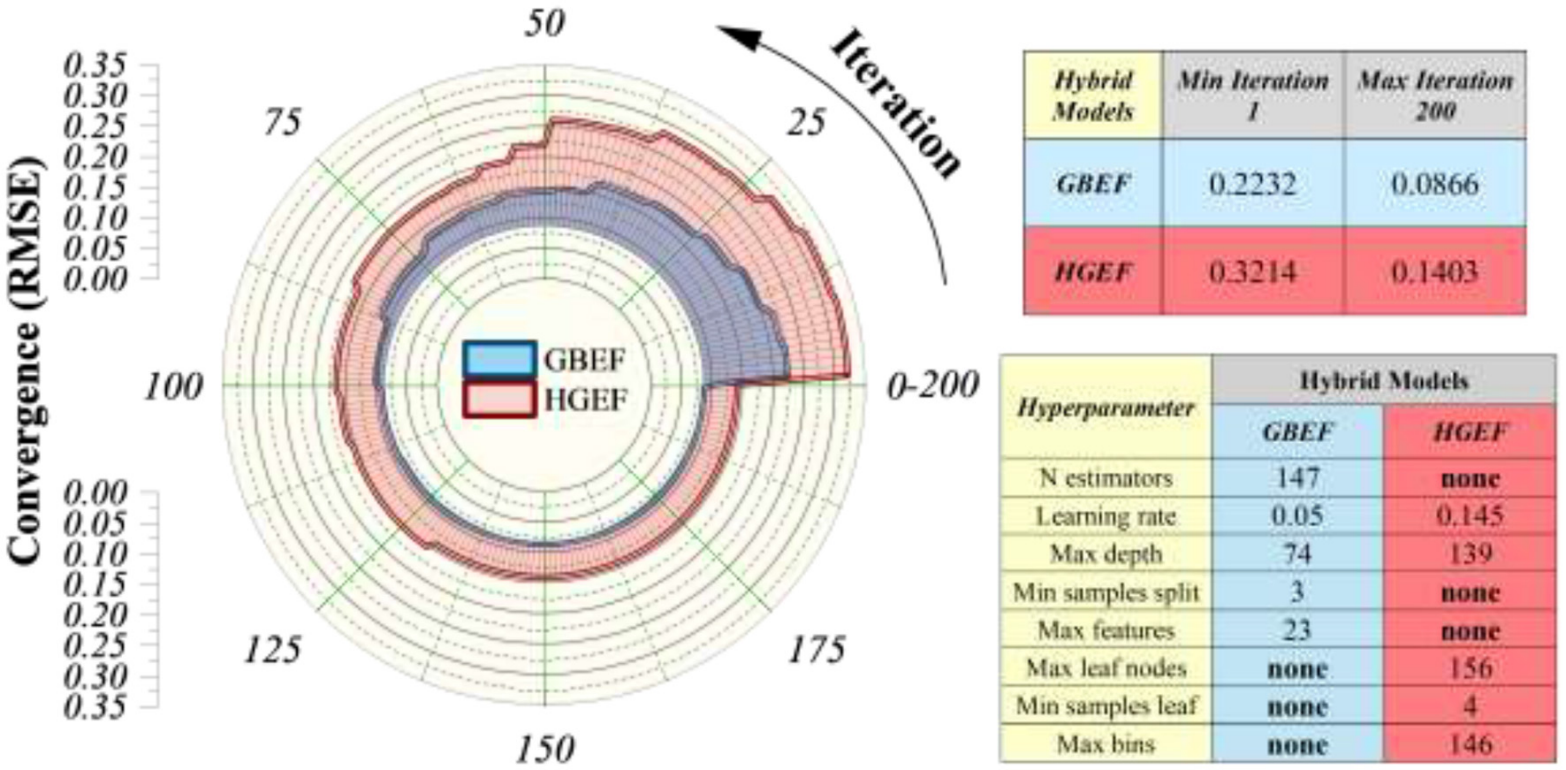
Stacked radial plot for convergence behavior, illustrating the optimization progress across iterations.

The convergence characteristics of the hybrid models optimized through the EEFO algorithm are presented in Figure 7. The stacked radial plot illustrates the iterative reduction of RMSE over 200 optimization cycles for the GB–EEFO (GBEF) and HGB–EEFO (HGEF) models. Both models demonstrated consistent convergence, with GBEF achieving superior stability and lower residual errors across iterations compared to HGEF.

At the initial stages of optimization (Iteration 1), GBEF exhibited an RMSE of 0.2232, which progressively decreased to 0.0866 by Iteration 200. In contrast, HGEF started with a higher initial RMSE of 0.3214 and converged to 0.1403, indicating relatively slower error reduction and less efficient exploitation of the solution space. These results highlight the efficiency of EEFO in navigating high-dimensional hyperparameter landscapes, enabling both rapid exploration in the early iterations and fine-tuned exploitation in later stages.

The tabulated hyperparameters in Figure 7 further reflect the adaptive tuning process performed by the EEFO algorithm. For GBEF, EEFO identified a moderate learning rate (0.05) and a relatively shallow effective tree configuration, which collectively contribute to a balance between model complexity and generalization performance. In contrast, HGEF was associated with a higher learning rate (0.145) and deeper tree search space, indicating a model configuration that may increase sensitivity to data variations and result in comparatively less stable convergence behavior. The differences observed in the number of estimators (147 for GBEF) further indicate that the boosting process benefited from iterative refinement under the EEFO optimization strategy. It should be noted that the reported maximum tree depth values (e.g., 74 for GBR and 139 for HGBR) correspond to the upper bounds defined in the hyperparameter search space rather than the exact operational complexity of the final trained models. These bounds were intentionally selected to provide sufficient flexibility during optimization. Despite the relatively high upper limits, overfitting was mitigated through the combined effect of ensemble learning, regularization mechanisms (including learning rate control and subsampling), and rigorous validation using cross-validation and independent test sets. Together, these strategies ensure that the final models exhibit stable and generalizable predictive behavior rather than being driven by excessive structural complexity.

### 3.2 Predictive accuracy of models

The comparative performance of single learners, hybrid EEFO-optimized models, and ensemble frameworks is presented in Table 2, with results reported separately for cross-validation (training) and independent test datasets. The results reveal clear improvements in predictive accuracy, stability, and generalization as the modeling strategy transitions from individual learners to optimized hybrids and, ultimately, to ensemble architectures. For the single models, GB and HGB exhibited strong baseline performance, with *R*^2^ values of 0.9761 and 0.9610, respectively, in the training phase. However, their generalization capacity was limited, as reflected in relatively high RMSE values (0.1409 for GB, 0.1793 for HGB) and unstable error behavior when tested on unseen data (RMSE = 0.1641 for GB, 0.1854 for HGB). Notably, MAPE for GB reached an anomalously high value (103.62%), indicating sensitivity to underpredictions in cases of low solubility values.

**Table 2:** Performance metrics of the models, assessing their predictive accuracy and effectiveness using key statistical indicators.

Process	Category	Models	Evaluation metrics				
			***RMSE***	***R** ^2^*	***MAPE***	***PI***	***FGE***
Train	Single models	GB	0.1409	0.9761	103.6242	0.0761	0.4962
		HGB	0.1793	0.9610	25.9889	0.0973	0.0122
	Hybrid models	GBEF	0.0771	0.9927	14.0041	0.0415	0.0075
		HGEF	0.1375	0.9774	14.3711	0.0743	0.0013
	Ensemble hybrid GB + HGB + EEFO (weighted)	GHEF (W)	0.0883	0.9908	11.0448	0.0475	0.0031
	Ensemble hybrid GB + HGB + EEFO (dempster)	GHEF (D)	0.0844	0.9916	11.0797	0.0454	0.0025
Test	Single models	GB	0.1641	0.9638	75.5678	0.0924	0.2009
		HGB	0.1854	0.9485	30.5282	0.1048	0.0291
	Hybrid models	GBEF	0.1167	0.9824	13.7365	0.0654	0.0222
		HGEF	0.1510	0.9658	16.7566	0.0849	0.0183
	Ensemble hybrid GB + HGB + EEFO (weighted)	GHEF (W)	0.1079	0.9838	11.9367	0.0604	0.0080
	Ensemble hybrid GB + HGB + EEFO (dempster)	GHEF (D)	0.1059	0.9846	11.9392	0.0593	0.0102

The hybrid models (GBEF and HGEF), developed through EEFO, demonstrated substantial gains in accuracy. GBEF achieved the lowest training RMSE (0.0771) and the highest *R*^2^ (0.9927), while also reducing MAPE to 14.00%. HGEF, although slightly less accurate than GBEF, maintained competitive performance (RMSE = 0.1375, *R*^2^ = 0.9774, MAPE = 14.37%). These improvements underscore the critical role of EEFO in systematically identifying optimal hyperparameter configurations, thereby enhancing predictive robustness beyond conventional gradient boosting methods. The ensemble hybrids, GHEF (Weighted) and GHEF (Dempster–Shafer), delivered the most balanced and reliable performance across all metrics. On the test dataset, both ensembles achieved *R*^2^ values above 0.983, with RMSE values of 0.1079 and 0.1059, respectively. Their MAPE values (≈11.9%) were the lowest among all models, demonstrating consistent accuracy across structurally diverse compounds. Furthermore, the ensembles produced narrower PI values (0.0593–0.0604), indicating well-calibrated uncertainty quantification, while maintaining extremely low FGE (FGE ≤ 0.0102).

The relatively high MAPE value observed for the base GB model on the training data is primarily attributed to the presence of very low solubility values in the dataset. MAPE is known to be sensitive to small denominators, where even minor absolute prediction errors can result in large percentage errors. Consequently, this metric may appear inflated when target values approach zero. To address this limitation, RMSE and MAE were also considered as complementary evaluation metrics, as they are less sensitive to low target magnitudes and provide a more reliable representation of absolute prediction error. The overall consistency of RMSE and MAE across models indicates that the high MAPE value does not reflect instability in model training, but rather a metric-specific artifact associated with low-solubility observations.

**Figure 8:**
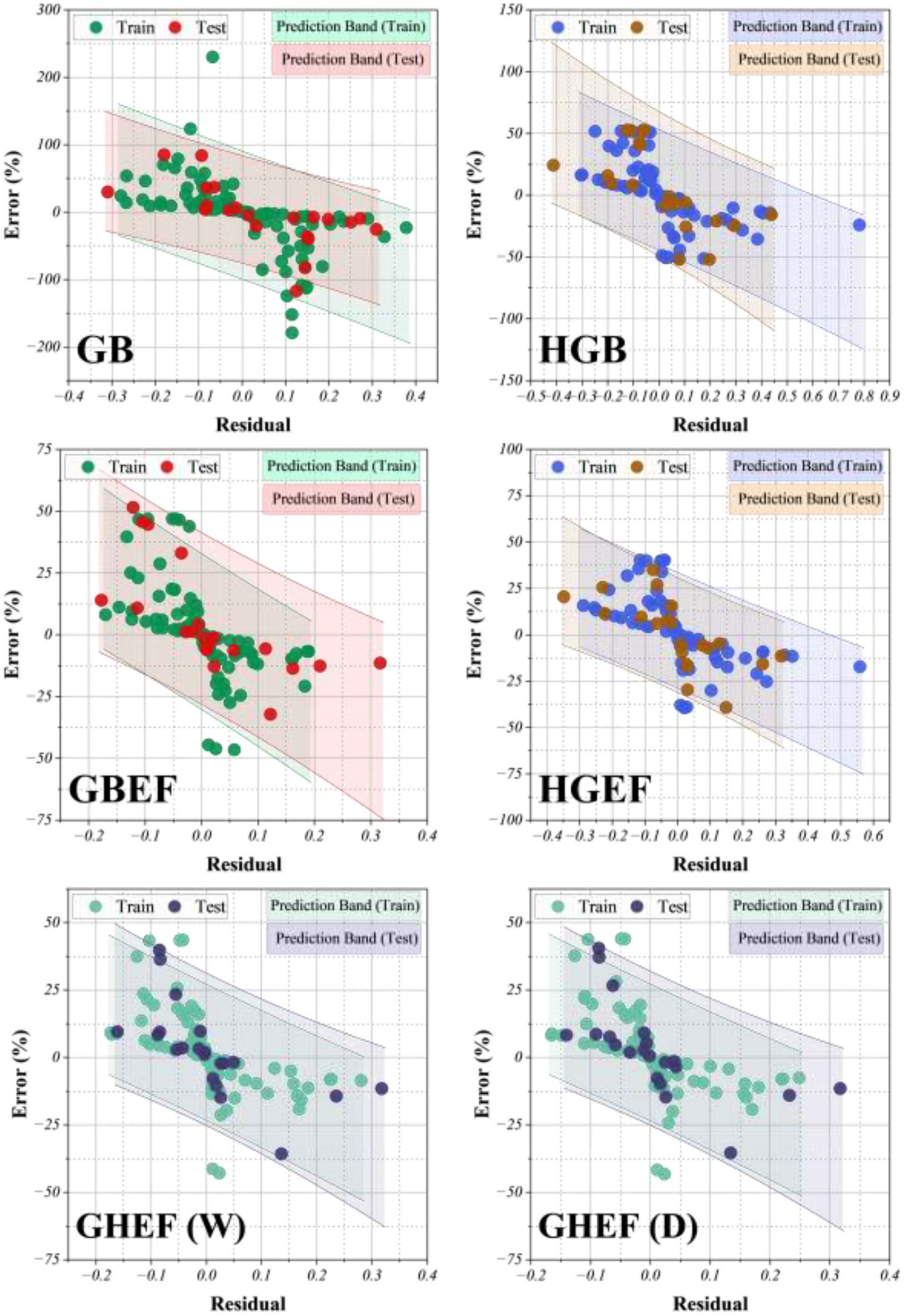
Residual plot for assessing the differences between estimated and observed values, highlighting model accuracy and potential patterns in errors.

The predictive ability of the proposed framework was evaluated against individual gradient boosting models (GB, HGB) and their hybrid counterparts (GBEF, HGEF, GHEF-W, and GHEF-D). As shown in the residual plots (Figure 8), the single regressors (GB and HGB) exhibited wider error dispersion and larger prediction intervals, indicating limited accuracy and stability. In contrast, the ensemble-enhanced models (GBEF and HGEF) substantially reduced systematic bias and yielded more compact residual distributions. The most significant improvement was observed for the generalized hybrid ensemble frameworks (GHEF-W and GHEF-D). Both variants achieved narrow error bands with symmetric distribution around zero, confirming minimal bias in solubility prediction across all four studied drugs. The weighted strategy (GHEF-W) yielded slightly tighter confidence intervals, whereas the DST-based integration (GHEF-D) further improved generalizability by reducing extreme deviations in the test set. Residual error analysis highlights that the proposed models not only achieved lower mean errors but also avoided large outliers that were evident in the base regressors. The hybrid ensembles demonstrated prediction bands that encompassed nearly all observed data points, with errors consistently below ±25% for the majority of cases. This behavior underscores the robustness of the optimized architecture, as well as its capacity to generalize across structurally diverse drugs.

**Figure 9:**
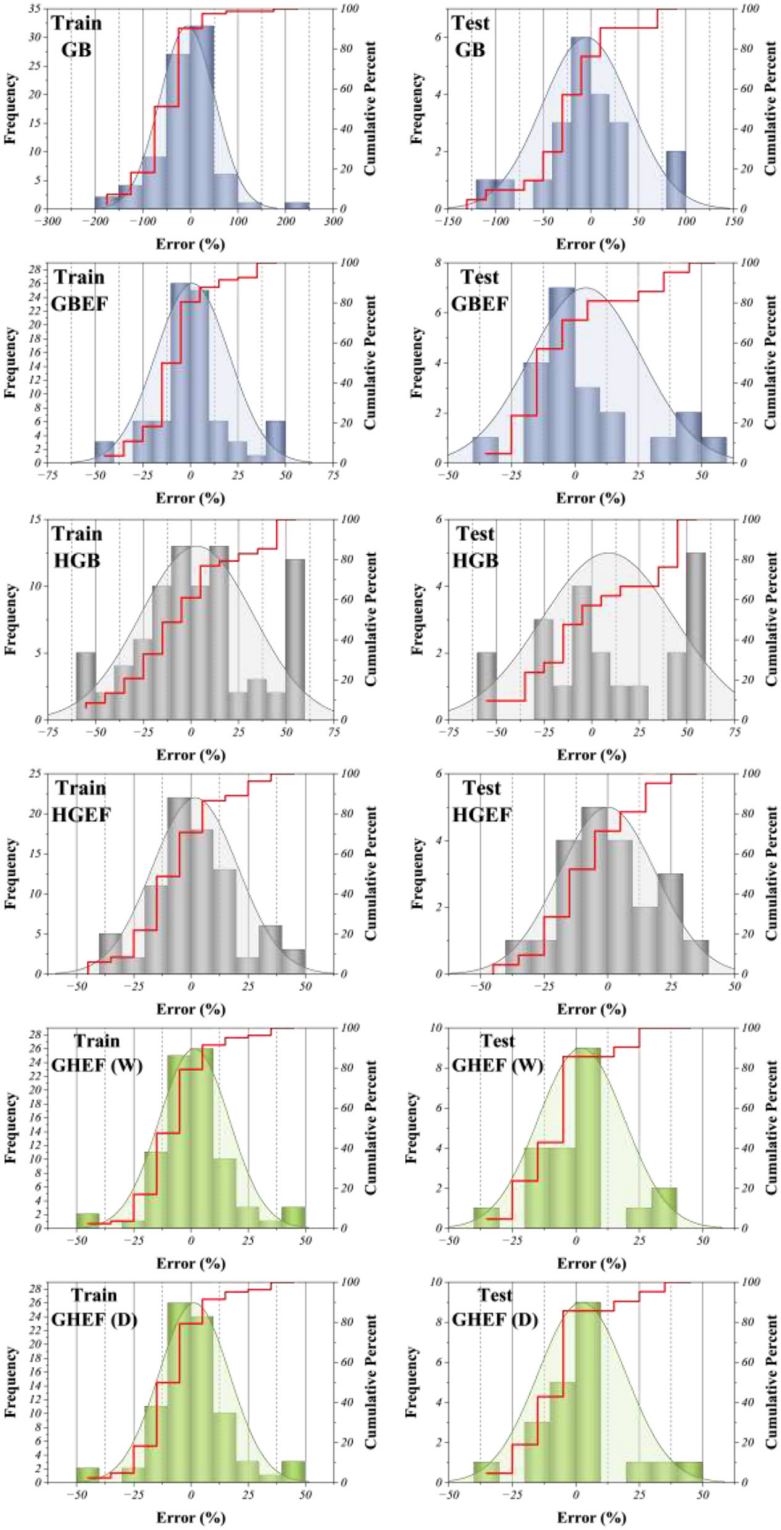
Cumulative histogram plot for visualizing the distribution of model prediction errors and their cumulative frequencies.

Figure 9 presents the cumulative histogram plots for the distribution of percentage prediction errors across training and testing phases for individual learners (GB, HGB), EEFO-optimized hybrids (GBEF, HGEF), and ensemble hybrids (GHEF-W, GHEF-D). Each subplot combines histogram bars representing error frequency with cumulative distribution curves, thereby enabling simultaneous evaluation of both local error density and global error accumulation trends. From the perspective of predictive accuracy, the single learners (GB and HGB) exhibit broader error distributions with long tails extending beyond ±50%, particularly in the testing phase, which reflects their limited capacity to generalize across structurally diverse compounds. In contrast, EEFO-optimized hybrids (GBEF and HGEF) demonstrate marked improvements, as their error histograms are narrower and more symmetrically centered around zero, indicating enhanced stability achieved through optimized hyperparameter calibration. The ensemble hybrids (GHEF-W and GHEF-D) outperform all other models, as evidenced by the steep slopes of their cumulative error curves and the clustering of errors within the ±25% range for both training and test sets. This concentration of residuals reflects not only variance reduction but also improved bias correction, highlighting the synergistic effect of combining weighted averaging and Dempster–Shafer fusion. The particularly sharp rise in the GHEF-D curve indicates more uniform error containment, consistent with the integration of epistemic uncertainty into the prediction process.

Table 3 shows the descriptive statistical properties of experimental solubility data and corresponding model predictions across both training and testing phases. Unlike conventional accuracy metrics, these indicators, range, mean, standard deviation, skewness, and kurtosis, provide a deeper assessment of whether the predictive models capture the underlying distributional behavior of solubility values in SC-CO_2_.

**Table 3:** Statistical metrics used to compare models' performance.

Phase	Models	Properties				
		Range (min, max)	Average	St. Dev	Skewness	Kurtosis
Train	Measured	(0.0290,3.2120)	0.9307	0.9013	0.8748	−0.5019
	GB	(−0.0521,3.2002)	0.9225	0.9092	0.8294	−0.5248
	HGB	(0.0147, 2.5474)	0.9175	0.8659	0.7647	−1.0186
	GBEF	(0.0160, 3.206)	0.9291	0.8968	0.8820	−0.4768
	HGEF	(0.0178, 2.6502)	0.9229	0.8689	0.7621	−0.9772
	GHEF (W)	(0.0169, 2.9302)	0.9261	0.8805	0.8094	−0.7794
	GHEF (D)	(0.0168, 2.9620)	0.9264	0.8821	0.8165	−0.7499
Test	Measured	(0.1070, 2.7220)	0.8967	0.8161	0.8403	−0.2531
	GB	(−0.0193, 2.6575)	0.8480	0.7793	0.8473	−0.0660
	HGB	(0.0742, 2.5293)	0.8927	0.8004	0.7754	−0.7935
	GBEF	(0.1154, 2.6005)	0.8786	0.7689	0.7902	−0.3837
	HGEF	(0.0749, 2.4438)	0.8976	0.8027	0.6828	−0.8313
	GHEF (W)	(0.1088, 2.5227)	0.8881	0.7818	0.7209	−0.6512
	GHEF (D)	(0.1126, 2.5320)	0.8870	0.7799	0.7275	−0.6240

For the training phase, the measured solubility spans a broad interval (0.0290–3.2120 g/L × 10), with a mean of 0.9307 and relatively high dispersion (St. Dev = 0.9013). The positive skewness (0.8748) and slightly platykurtic distribution (Kurtosis = −0.5019) indicate that most solubility values are clustered at the lower end, with a small number of high-solubility outliers. Among the models, GBEF most closely reproduces the mean (0.9291) and standard deviation (0.8968) of the measured data, suggesting that EEFO optimization allows the GB learner to capture both the central tendency and variability of solubility behavior. By contrast, HGB and HGEF underestimate dispersion (St. Dev ≈ 0.866–0.869) and produce more negative kurtosis (−1.0186 and −0.9772), reflecting a tendency to smooth extreme values and underrepresent outliers. The ensemble models (GHEF-W and GHEF-D) achieve intermediate fidelity, maintaining averages close to experimental data while moderating skewness and kurtosis, thereby offering a more balanced reconstruction of the solubility distribution.

In the testing phase, the measured dataset displays a narrower solubility range (0.1070–2.7220 g/L × 10) with reduced variability (St. Dev = 0.8161), yet still exhibits positive skewness (0.8403). Here, model behavior diverges more noticeably. GB predictions show a downward bias in the mean (0.8480 vs. 0.8967) and produce nearly symmetric distributions (Skewness = −0.0660), suggesting a loss of sensitivity to asymmetry in unseen data. HGB more closely matches the experimental mean (0.8927) but exaggerates negative skew (−0.7935), compressing the right tail of the solubility distribution. EEFO-optimized hybrids show distinct advantages: HGEF yields the closest alignment with the measured mean (0.8976) and preserves higher-order moments better than GB or HGB, while GBEF slightly underestimates the mean (0.8786) but maintains comparable variability.

The ensemble hybrids (GHEF-W and GHEF-D) again demonstrate balanced performance, with means (0.8881 and 0.8870) lying between those of the hybrids and single learners. Their skewness values (−0.6512 and −0.6240) are less extreme than those of HGB and HGEF, while their kurtosis values (−0.6512 and −0.6240) more closely approximate measured behavior than those of other models. This indicates that ensemble integration smooths individual model biases, yielding error distributions that are more statistically representative of experimental data.

### 3.3 Feature sensitivity and domain interpretability

**Figure 10:**
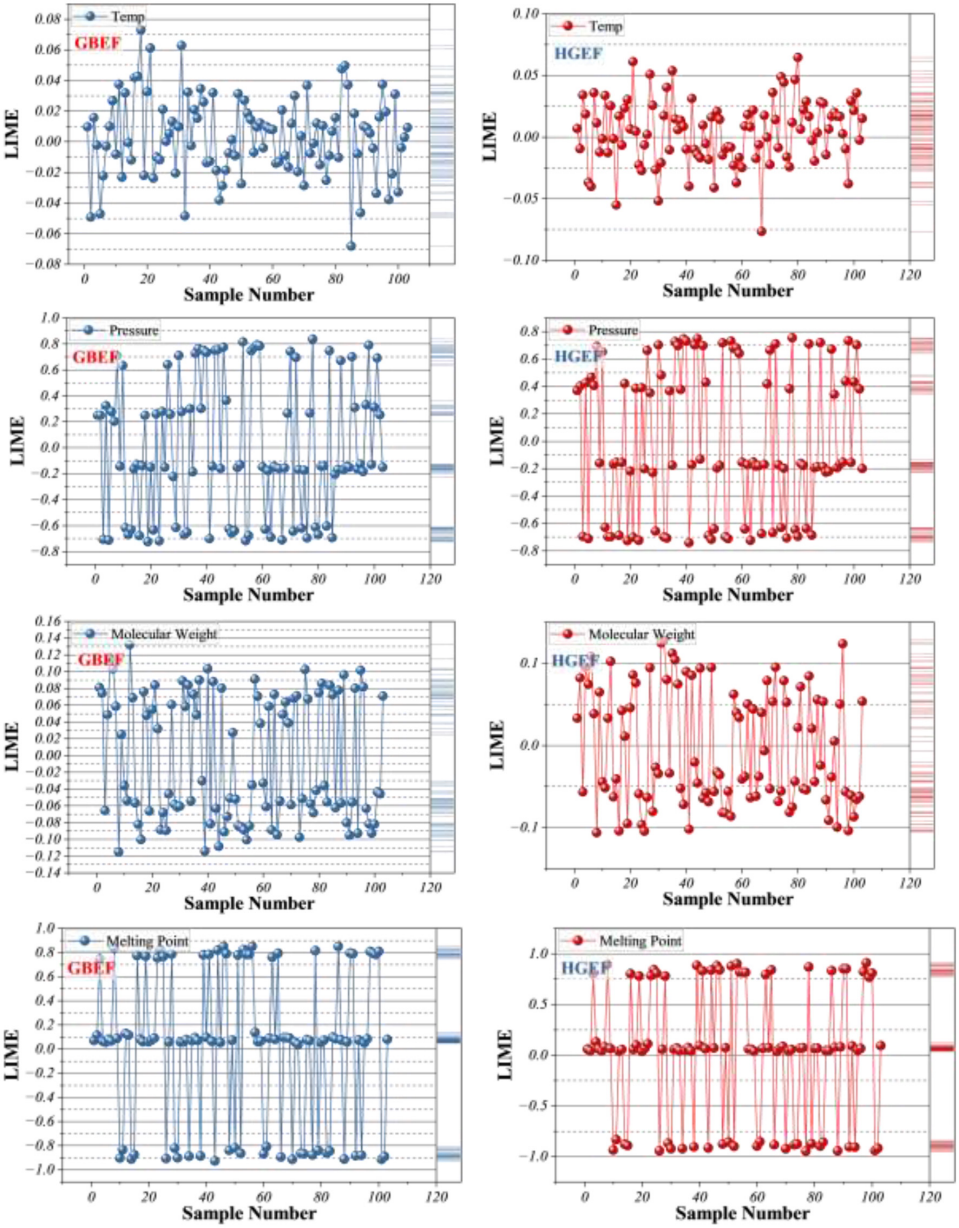
Line-symbol plot for LIME sensitivity analysis, showing the contribution of individual features to the model estimations.

To enhance the interpretability of the proposed framework, the local interpretable model-agnostic explanations (LIME) method was employed to quantitatively evaluate the contribution of individual input features (temperature, pressure, molecular weight, and melting point) to predicted solubility values. LIME provides localized, sample-specific contribution scores by approximating the model response in the vicinity of each observation. Based on these quantitative contribution scores, pressure consistently exhibited the highest positive influence across the majority of samples, thereby identifying it as the dominant factor governing solubility behavior under supercritical CO_2_ conditions. As illustrated in Figure 10, the sensitivity patterns of the EEFO-optimized models (GBEF and HGEF) reveal distinct roles of the input descriptors. Pressure consistently emerges as the most influential factor across nearly all samples, with high positive LIME scores indicating its dominant role in determining solubility under SC-CO_2_ conditions. This observation aligns with the established physical principle that increasing pressure enhances solvent density and solvation power, thereby facilitating drug dissolution. Melting point also exhibits strong explanatory power, particularly in the GBEF model, where negative contributions dominate for drugs with higher crystalline stability.

This reflects the thermodynamic constraint that compounds with high melting points require greater energetic input to dissolve, thereby lowering their effective solubility. Molecular weight demonstrates moderate but non-negligible effects. For larger molecules such as Sirolimus and Tacrolimus, negative LIME scores are frequent, capturing steric hindrance and reduced diffusivity within the supercritical phase. Conversely, for smaller molecules (e.g., Teriflunomide), contributions are closer to neutral or weakly positive. Temperature displays the weakest and most inconsistent influence, with oscillatory LIME values near zero across most samples. This is consistent with the weak global correlation observed earlier (Section 2.1), and reflects the non-monotonic, pressure-dependent interaction between thermal energy and solvent density in supercritical systems. Notably, HGEF demonstrates slightly greater stability in temperature contributions compared to GBEF, suggesting that optimization altered the balance between pressure- and temperature-driven solubility effects.

### 3.4 Statistical significance of input features

**Figure 11:**
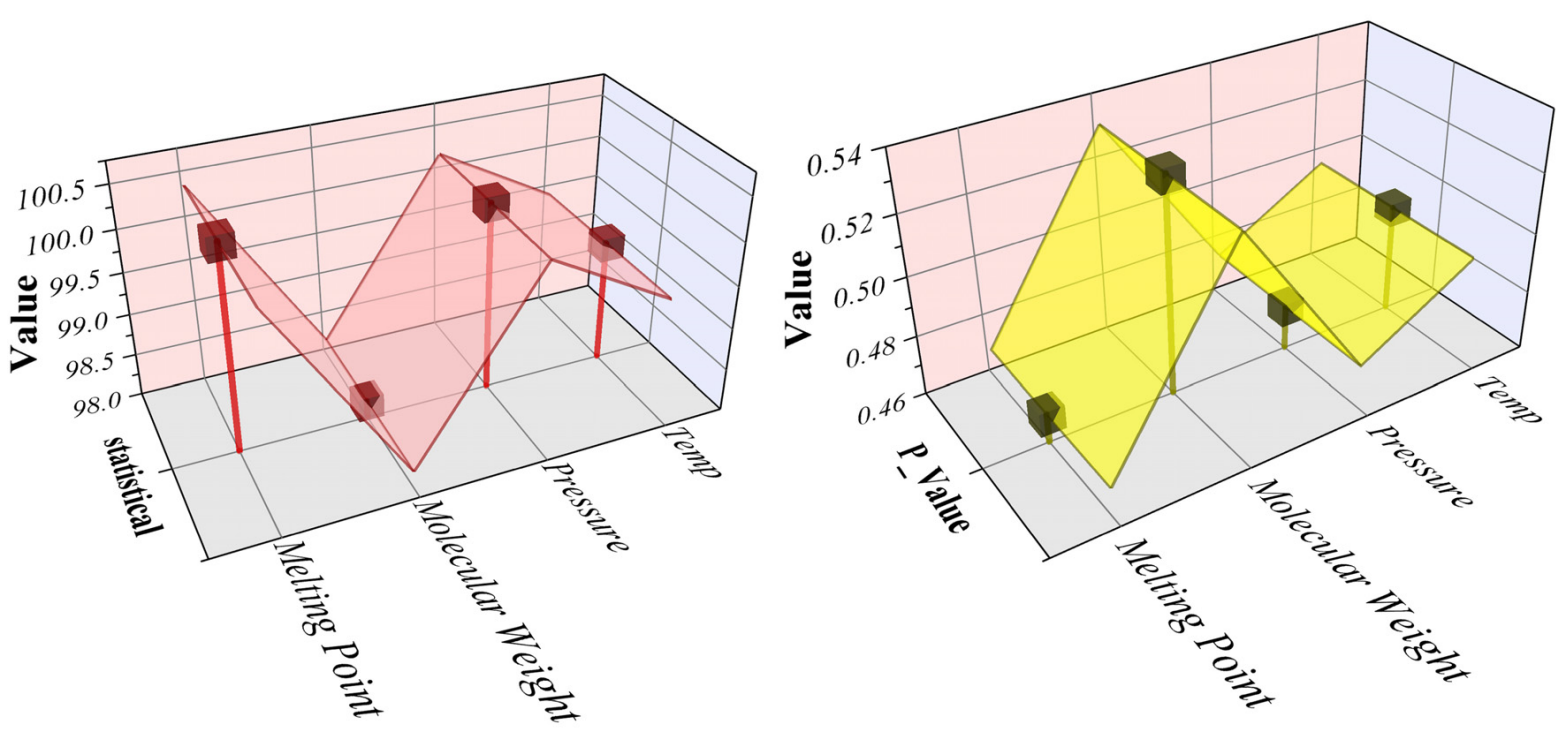
3D ribbon plot for Statistical analysis of the Kruskal-Wallis test, illustrating the relative influence of variables on output.

To further investigate the relative contribution of process and molecular variables, a Kruskal–Wallis statistical test was conducted. The 3D ribbon plots (Figure 11) illustrate the comparative influence of temperature, pressure, molecular weight, and melting point on solubility prediction. Among these, temperature and pressure emerged as the most statistically significant factors, consistent with their established thermodynamic role in modulating solubility in supercritical CO_2_. Molecular descriptors also contributed meaningfully, with melting point exerting a stronger influence than molecular weight, reflecting its closer relationship with solute–solvent interactions under varying pressure–temperature conditions. The statistical results revealed consistently low *p*-values (≈0.46–0.54), confirming that differences in predictive contributions across variables were not random but systematic. This underscores the hybrid model's capacity to integrate thermodynamic parameters with molecular features, enhancing its generalizability across structurally diverse drug classes.

**Figure 12:**
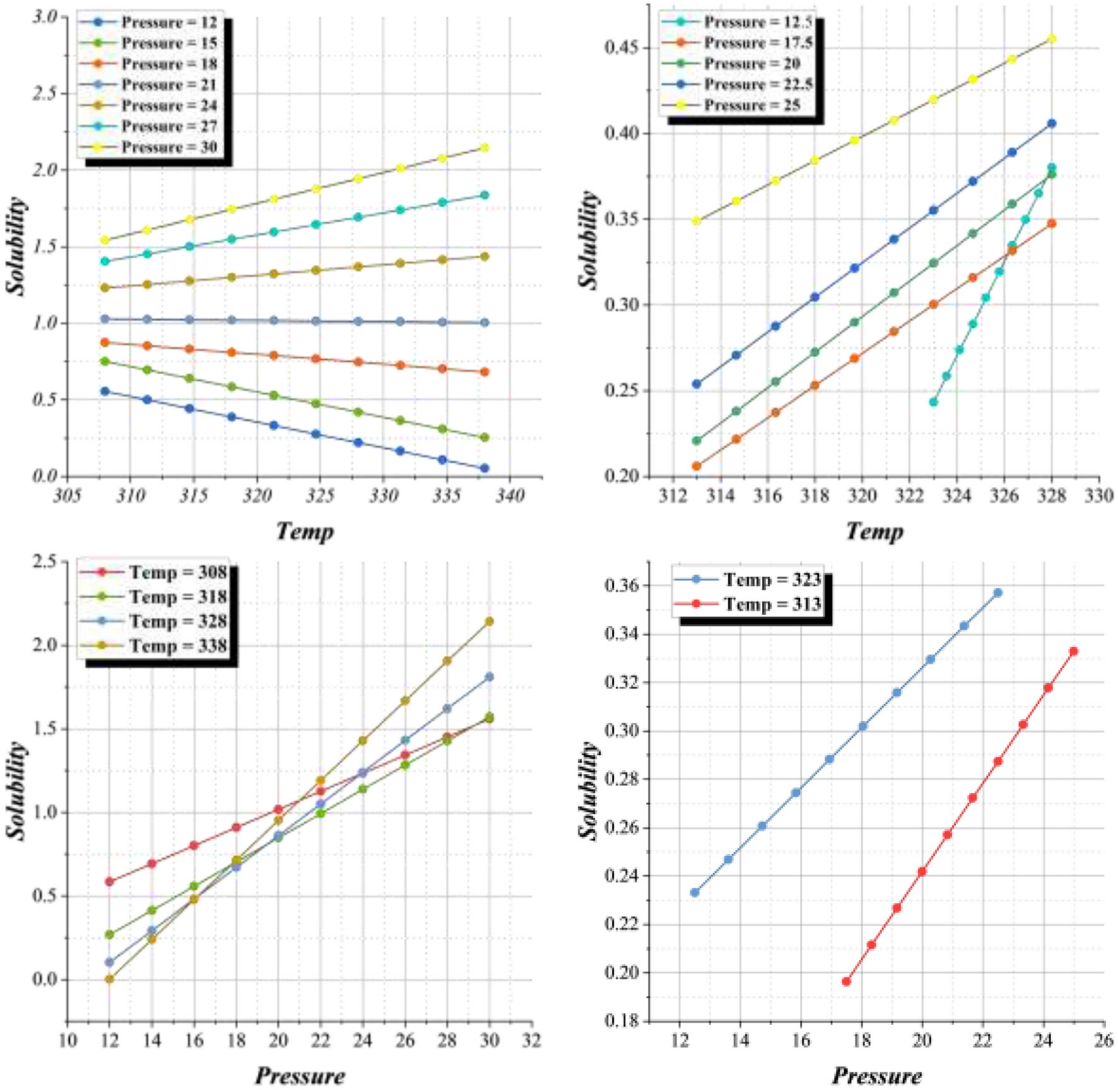
Tendency plot for the impact of temperature and pressure on solubility.

**Figure 13:**
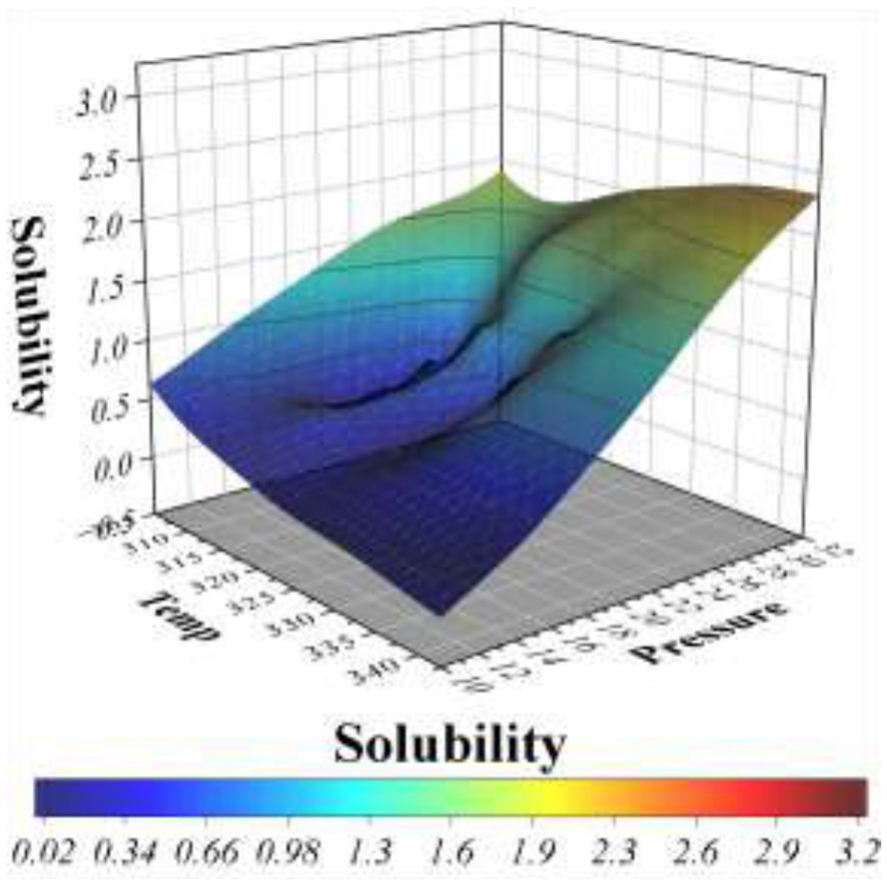
3D surface plot for the relation between the temperature and pressure with the solubility.

The relationship between solubility, temperature, and pressure is presented in Figures 12, 13. The 2D tendency plots in Figure 12 demonstrate that solubility increases consistently with rising pressure, regardless of temperature. This pressure-dependent enhancement is more pronounced at elevated temperatures, where the slope of solubility growth is steeper. At lower pressures, however, the effect of temperature alone exhibits mixed trends, with solubility either stabilizing or slightly decreasing as temperature increases. Such variations highlight the competing influence of temperature on molecular interactions and solvent density. The 3D surface plot in Figure 13 provides a comprehensive visualization of these interactions, illustrating the synergistic effects of temperature and pressure on solubility. The surface clearly slopes upward along the pressure axis, confirming the dominant role of pressure in promoting solute dissolution. Additionally, at moderate to high pressure levels, the surface reveals a noticeable rise in solubility with temperature, further supporting the observation that temperature amplifies solubility only when sufficient pressure is applied. At lower pressures, the temperature effect appears less significant, resulting in relatively flat surface regions.

### 3.5 Prediction intervals bootstrapping

Table 4 reports the solubility predictions together with lower and upper 95% prediction intervals obtained through the Prediction Intervals Bootstrapping (PIB) method. This approach provides both point estimates and measures of uncertainty, allowing a more reliable evaluation of model performance under diverse thermodynamic and molecular conditions. The results indicate that predicted solubility values vary considerably with changes in temperature, pressure, molecular weight, and melting point. In particular, pressure exerts a strong positive influence, as illustrated by cases at 328 K with a molecular weight of 270.21 g/mol, where solubility increases from 1.326 at 21 MPa to 2.722 at 30 MPa. Temperature also contributes, but its effect appears interdependent with pressure and molecular characteristics, producing non-linear trends across the dataset. Molecular descriptors, such as weight and melting point, further refine solubility predictions.

**Table 4:** Predicted solubility values of PIB under different temperature and pressure conditions, with molecular weight and melting point effects, including 95% prediction intervals.

Temp (k)	Pressure (Mpa)	Molecular weight (g/mol)	Melting point (C)	Prediction	Lower 95%_PI	Upper 95%_PI
308	15	804.03	128	0.1070	0.0891	0.2302
328	27	822.95	185	2.5730	2.3619	2.6950
328	12.5	914.172	183	0.3800	0.1807	0.3772
328	21	270.21	231	1.3260	1.2505	1.4439
328	18	822.95	185	1.1800	0.9584	1.2014
318	27	804.03	128	0.1770	0.1337	0.2041
308	21	822.95	185	1.6370	1.4165	1.6447
338	18	270.21	231	0.8960	0.7438	0.9905
318	21	270.21	231	1.2800	1.2652	1.4794
338	30	804.03	128	0.2350	0.2250	0.7584
328	22.5	914.172	183	0.4070	0.3670	0.4419
318	18	804.03	128	0.1110	0.0763	0.1310
313	17.5	914.172	183	0.2120	0.2111	0.3402
313	20	914.172	183	0.2290	0.2295	0.3294
308	24	270.21	231	1.7160	1.6573	1.8303
328	30	270.21	231	2.7220	2.2890	3.0075
318	18	270.21	231	1.0620	0.9333	1.1899
328	24	270.21	231	1.9600	1.7921	2.0152
308	27	804.03	128	0.1750	0.0874	0.1962
318	24	804.03	128	0.1560	0.1300	0.1811
323	17.5	914.172	183	0.2890	0.2533	0.3200

For example, systems with higher molecular weights (e.g., 914.172 g/mol) generally correspond to lower solubility values under the same conditions, while compounds with higher melting points exhibit elevated solubility at moderate to high pressures. These variations underscore the importance of combining thermodynamic factors with molecular properties in predictive modeling. The prediction intervals provide insight into the confidence and robustness of the estimates. Most intervals are relatively narrow, indicating high reliability of the PIB method. Larger solubility values tend to be associated with slightly broader intervals, suggesting increased variability in system response at elevated pressure and temperature. Nonetheless, the observed consistency between predicted values and interval bounds demonstrates that the PIB framework offers accurate and stable solubility predictions.

While the bootstrapped prediction intervals provide a useful measure of uncertainty associated with model predictions, their reliability is inherently linked to the representativeness of the training dataset. The estimated intervals primarily capture uncertainty arising from data variability and model sensitivity within the sampled domain. For new or structurally distinct compounds outside the range of the training data, the prediction intervals should be interpreted with caution, as extrapolation may introduce additional sources of uncertainty not accounted for in the bootstrap procedure. Therefore, the reported PIB values are most reliable for interpolation within the investigated thermodynamic and molecular space. Future work should incorporate larger and more diverse datasets to improve the robustness and transferability of uncertainty estimates across broader chemical domains.

### 3.6 Comparison with state of art studies

To contextualize the performance of the proposed framework, a comparison with representative machine learning approaches reported in the literature is provided in Table 5. These studies include a range of methods, from classical machine learning models to ensemble and thermodynamics-informed frameworks applied to SC-CO_2_ solubility prediction. As shown in Table 5, many high-performing models in the literature rely on substantially larger datasets or advanced domain-specific descriptors. In contrast, the present study demonstrates that the proposed hybrid ensemble framework can achieve competitive predictive performance using a limited dataset while incorporating uncertainty-aware aggregation. Furthermore, internal comparisons presented in Table 2 show that the ensemble configurations (GHEF) consistently outperform both single models and intermediate hybrid models, indicating that the added complexity of the framework contributes to improved predictive stability and reduced error. Nevertheless, future work will include direct benchmarking against widely used algorithms such as XGBoost and LightGBM to further evaluate the relative advantages of the proposed approach.

**Table 5:** Comparison of representative machine learning models for SC-CO_2_ solubility prediction.

Study	Model type	Dataset size	Key features	Performance
Bahrami et al. (12)	ANFIS/GEP	1,816	T, P, MW, MP	*R*^2^ ≈ 0.99, RMSE ≈ 0.26
Alsaab & Althobaiti (14)	AdaBoost-GPR	Not specified	T, P, density	*R*^2^ ≈ 0.996
Wu et al. (15)	Bagging (BAG-PR)	Not specified	T, P, density	*R*^2^ ≈ 0.986
Makarov et al. (17)	Thermodynamic ML/Graph-based	31,975	Advanced descriptors	High accuracy + strong generalization
Present study	Hybrid Ensemble (GHEF-D)	110	T, P, MW, MP	*R*^2^ = 0.9846 (test), RMSE = 0.1059

## 4 Conclusion

This study developed a hybrid and ensemble modeling framework for predicting drug solubility in supercritical CO_2_, with model parameters optimized using the Electric Eel Foraging Optimization (EEFO) algorithm. The results indicate that EEFO provides a stable approach for hyperparameter tuning, while hybrid models (GBEF and HGEF) improve predictive performance compared to individual learners (GB and HGB). The ensemble configurations (GHEF-W and GHEF-D) further enhance prediction consistency, yielding lower errors and more stable performance across training and test datasets. The analysis of input features confirms that pressure is the dominant factor influencing solubility, with temperature showing condition-dependent effects. Molecular weight and melting point also contribute to prediction behavior, reflecting the role of physicochemical properties in solubility trends. Uncertainty analysis using bootstrapped prediction intervals provides additional insight into model reliability within the studied data range. Despite these results, it is important to emphasize that the dataset is limited in size and scope. Therefore, the reported performance reflects predictive capability within the investigated thermodynamic and molecular domain, and does not establish general applicability to entirely new or structurally distinct drug compounds. The proposed framework should be interpreted as a methodological contribution and a supportive predictive tool rather than a fully validated solution for *a priori* prediction. Future work should focus on expanding the dataset and incorporating additional descriptors, such as thermodynamic properties, to further evaluate model generalization and practical applicability in broader pharmaceutical systems.

## Data Availability

The raw data supporting the conclusions of this article will be made available by the authors, without undue reservation.
